# In vitro and ex vivo models of the oral mucosa as platforms for the validation of novel drug delivery systems

**DOI:** 10.1177/20417314241313458

**Published:** 2025-02-11

**Authors:** Robyn A Macartney, Abijit Das, Atina G Imaniyyah, Annabelle TR Fricker, Andrew M Smith, Stefano Fedele, Ipsita Roy, Hae-Won Kim, Dongjoon Lee, Jonathan C Knowles

**Affiliations:** 1 Division of Biomaterials and Tissue Engineering, University College London (UCL) Eastman Dental Institute, London, UK; 2 Institute of Tissue Regeneration Engineering (ITREN), Dankook University, Cheonan, Republic of Korea; 3 Department of Nanobiomedical Science &BK12 NBM Global Research Center for Regenerative Medicine, Dankook University, Cheonan, Republic of Korea; 4 School of Chemical, Materials & Biological Engineering, Faculty of Engineering, University of Sheffield, Sheffield, UK; 5 Department of Microbial Diseases, UCL Eastman Dental Institute, University College London, London, UK; 6 UCL Eastman Dental Institute, University College London, London, UK; 7 Insigneo Institute for in silico Medicine, University of Sheffield, UK; 8 College of Dentistry, Dankook University, Cheonan, Republic of Korea

**Keywords:** Oral mucosa, tissue model, drug discovery, in vitro, ex vivo

## Abstract

The benefit of complex 3D models to facilitate the robust testing of new drugs and drug delivery systems during the developmental stages of pharmaceutical manufacturing has recently become distinguished within the field. Recognition of this need by the pharmaceutical industry has provided a motivation for research into the development of reliable complex models for use in drug delivery, biomaterials, and tissue engineering. Both 3D in vitro and ex vivo models can enhance drug-testing and discovery prospects over the more traditionally used 2D, monolayer culture systems and animal models. Despite the widespread acceptance that 3D tissue modelling is advantageous in this field, there remains a lack of standardisation in the models throughout literature. This article provides an extensive review of current literature on in vitro, and ex vivo models of the oral mucosa for drug delivery applications; the advantages, limitations, and recommendations for future development of improved models for this application.

## Introduction

The importance of developing complex in vitro tissue models as drug testing platforms has become highlighted during recent years. These models offer more physiologically relevant environments for testing new drugs and drug delivery systems (DDS). Whilst traditional high-throughput methods of drug screening using tissue culture plastic matrices have been widely accepted for decades, several pharma companies have now shown acknowledgement of the need for more physiologically relevant platforms to boost drug discovery prospectives. A major advantage of developing these in vitro tissue models is the potential to minimise reliance on in vivo animal studies and help research adhere to the 3 R’s of animal testing. Thus, dramatically reducing the ethical concerns and costs associated with drug development. Additionally, in some cases in vitro models are considered superior to in vivo models due to the use of human cells, increasing the transferability compared to tests conducted in different species. Whilst these models have many advantages, researchers have described a range of limitations of in vitro and ex vivo models which should be addressed. These include problems with long term maintenance of viability during extended periods of culture and complex fabrication methods which lack standardisation.^1,2^

The development, optimisation and characterisation of oral mucosal models has been subject to increasing interest over the past decade. Several applications for such models have been described, namely biological characterisation of biomaterial interaction with the oral mucosa, modelling of oral infection/disease states and evaluation of DDSs.^3–5^ Many studies have reported the development of such models utilising a range of scaffolds, cell sources, seeding techniques and culture conditions. Within this review we will focus on the development of in vitro oral mucosal models for testing of DDSs. The designed model should mimic as closely as possible the native environment in which the DDS will be utilised; therefore the complex in vitro model should be tailored to the specific final application. This may introduce complexities within models for testing of DDSs designed to treat infections or trauma injuries of the oral mucosa itself as these disease states should be considered during the model development. Whereas, for a DDS designed for systemic delivery via the oral mucosal route an in vitro model of normal oral mucosal state may be appropriate.

Several advantages pertaining to the transmucosal oral delivery of drugs has led to increased interest in these kinds of DDSs. Bypass of the hepatic first-pass metabolism is frequently highlighted as a key advantage for the use of transmucosal drug delivery.^6^ Often, the bioavailability of drugs is greatly reduced due to metabolic action in the liver, this extreme reduction in the amount of active drug emerging from the liver to the circulatory system may be avoided via transmucosal delivery.^7^ Additional advantages include, easy and self-administered treatment, a range of dosage forms and rapid onset of action with the possibility to incorporate sustained release mechanisms.^8,9^ Despite the multiple advantages described here these DDSs do not come without obstacles. A universal obstacle with the development of these DDSs is related to the limitation of maximum dosage which can be administrated with a single treatment.^10^ Many of the other concerns are specific to certain patient groups. The oral transmucosal route is not suitable for patients suffering from frequent vomiting or those suffering dry mouth, as they may experience poor drug dissolution, leading to reduced adsorption through the mucosa.^11^ Conversely excessive saliva production, which can be common with neurological diseases, could cause a wash-out effect which also impairs mucosal adsorption.^12^ In young and elderly patients these DDSs may also pose choking risks.^13^ This concern may also come into play during periods of unconsciousness for patients admitted in hospital or other care settings.

## Drug delivery via the oral mucosa

**Figure 1.**
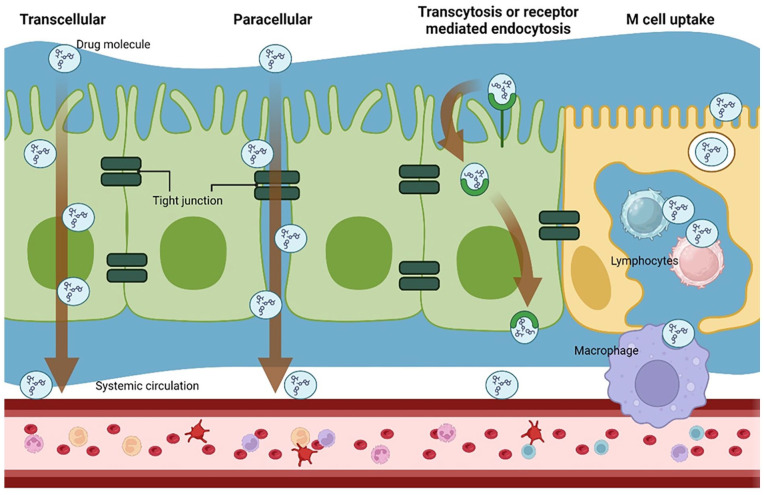
Pathways for therapeutic drug absorption via the oral route.

Among the various drug delivery routes, the oral pathway has attracted the most attention due to its unique advantages, including sustained and controllable delivery, ease of administration, feasibility for solid formulations, patient compliance and an intensified immune response in the case of vaccines.^14–16^ The oral mucosa serves as a versatile interface for drug delivery, offering several unique features that aid in the efficient and effective absorption of medications.^17,18^ One key advantage lies in its expansive surface area covering 170 cm^2^, encompassing diverse regions such as the buccal, sublingual, and gingival mucosa. This extensive coverage presents abundant opportunities for drug absorption, enabling swift and effective uptake of medications administered via the oral mucosa.^6^ This large surface area contributes to enhanced drug bioavailability, fostering optimal therapeutic outcomes.^17,19,20^ Furthermore, drug molecules trapped within mucus are protected against the shear stresses caused by flowing gastric juices.^21^ Orally administered drugs can be absorbed in four types of pathways: transcellular (intracellular), paracellular (intercellular), carrier-mediated transcellular and facilitated transport (Figure 1).

Among these pathways, the passive, transcellular and paracellular, pathways are the main mechanism. The choice of absorption route is dependent on the hydrophilic, hydrophobic or amphiphilic characteristics. Larger hydrophobic drug molecules typically prefer transcellular routes, while smaller hydrophilic molecules favour paracellular routes.^19^ The hydrophobic pathway utilises the paracellular lipid domains, while the hydrophilic pathway takes advantage of the fluid channels connected to the polar head regions of proteins and lipids. Most medications can diffuse via both pathways at the same time, however the route with lowest levels of resistance to penetration is typically preferred.^22,23^ These combined features of the oral mucosa render it an extremely versatile anatomical site for the delivery of a wide range of therapeutics. However, in comparison with other routes, the absorption mechanism for drugs via the oral mucosal pathway is more complex and faces several barriers. The barriers to oral mucosal drug absorption can be divided into two types: (i) metabolic and (ii) physical. With both barriers the extent to which they contribute to inhibition of drug movement are drug dependent. Additionally, the physical barrier to drug absorption is a function of the physicochemical properties of the mucosal membrane, specifically concerning the individual layers which constitute the oral mucosae.

## Features contributing to the effectiveness of drug delivery via the oral mucosa

The mucus layer covering the epithelium is the first structure encountered by a drug or DDS prior to absorption.^19,24,25^ Mucus plays a dual role in the absorption and desorption of medications administered orally. Two distinct overlaying layers typically make up the mucosal layer: an inner, firmly adhering layer and an outer, loosely adherent layer. The narrower inner mucus, a make-up of glycoproteins, glycolipids, and cell-bound mucin, is known to aid in drug absorption and improve uptake efficiency.^19^ The thicker mucus on the outside is a barrier that prevents medications and other molecules from moving freely. It acts as a selective filter to molecules and particles, keeping them from penetrating mucosal tissues’ epithelial surface. Depending on the type of drug carrier and the specific drugs involved, mucin can either enhance or decrease drug absorption.^26^ Its physicochemical characteristics might affect how DDSs ultimately behave and are delivered in mucosal tissues.^19,21^ Charged molecules, for example, interact with mucin through various mechanisms such as electrostatic attraction, hydrogen bonding, or hydrophobic interactions, which can hinder their transport through the buccal mucosa.^27,28^

The presence of mucus helps protect drug molecules against the shear stresses caused by flowing gastric juices by trapping them.^19^ Mucus acts as a powerful barrier that may be the primary obstacle to drug absorption by establishing an adhesive, unstimulated, viscoelastic layer next to the epithelial surface. It is designed to effectively trap and quickly remove microorganisms and foreign particles from unprotected epithelial surfaces. Mucus is constantly secreted to keep pathogens out of the body and to lubricate the epithelium’s surface as foreign objects pass through. This shortens the residence time of particles that are unable to pass through the GI mucus’s weakly adherent layer.^29^ Through several hypothesised processes, such as size exclusion, electrostatic and hydrophobic interactions, hydrogen bonding, and other bonding interactions, mucus controls permeability to substances and elements. Mucus’ physicochemical characteristics, including pore size, charge, ionic strength, viscoelasticity, and pH primarily control these pathways.^21^

Small molecules have been observed to freely diffuse across the mucus barrier, while larger macromolecules such as globular proteins are unable to penetrate it, suggesting that pore size may limit mucus permeability. Studies have indicated a decrease in particle mobility with increasing size,^30^ supporting this notion. However, size alone does not dictate permeability; larger virus-like particles have been found to diffuse more readily in human cervical mucus compared to smaller ones. Moreover, immunoglobulins form low-affinity bonds with mucins, diminishing their diffusivity within mucus. Additionally, research highlights the increased diffusivity of neutral polyethylene glycol-coated particles in mucus compared to uncoated ones,^31^ hinting at other filtration mechanisms like electrostatic or hydrophobic interactions, primarily due to negatively charged and hydrophobic regions in mucin fibres.^21^ The presence of glycoproteins and lipids in mucus forms a protective barrier over epithelial cells, enhancing the hydrophobic properties of intestinal linings. Studies indicate that materials with greater hydrophobicity, such as polystyrene and polyhydroxybutyrate, are absorbed more effectively by specific parts of the intestine compared to less hydrophobic materials. This preference for hydrophobic materials is attributed to their ability to adhere more readily to the intestinal surface, prolonging contact duration and thereby increasing absorption potential.^29^

To ensure effective drug absorption, molecules must swiftly traverse the mucus barrier to reach the underlying epithelium while avoiding rapid clearance and degradation. Achieving this requires optimal interaction between drug particles and biological surfaces. Drug particles must possess specific surface characteristics to navigate the mucus barrier without inhibition by mucin fibres. Mucin, a key component of the mucus barrier, is released by mucous cells in submucosal glands and goblet cells in the surface epithelium.^32^ The viscoelastic properties of oral mucus allow particles to adhere to the mucosal surface, providing a reservoir for drugs and facilitating prolonged contact with the mucosal epithelium.^29,32^ This prolonged contact enhances drug absorption and bioavailability, leading to improved therapeutic outcomes.^21^ While mucins primarily dictate the viscoelasticity of mucus, other components such as DNA, lipids, salts, and proteins also play a role in this aspect. Various interactions, including physically entangled non-covalent bonds and stronger covalent disulphide bonds between mucin fibres and other components, further shape the viscoelastic properties of mucus.^21^

Since mucin contains negative charges, opposing electric charges are needed to lengthen the period that particles are in the system and, as a result, boost drug absorption.^32^ For example, cationic mucoadhesive oligomers/polymers (e.g. chitosan) may interact electrostatically to limit mucous’ ability to complex cationic peptides.^33^ However, other investigations have shown that anionic polymers work to improve mucosal adherence.^34–37^ Studies conducted in the lower gastrointestinal (GI) tract regarding the interaction between nanoparticles and the mucus layer highlighted that, cationic nanoparticles were found to experience electrostatic repulsion with the negatively charged mucins present in the mucus layer. This repulsion impedes the movement or transport of the cationic nanoparticles through the mucus layer. In contrast, anionic nanoparticles were observed to diffuse more easily among the mucus networks. This is attributed to reduced electrostatic interaction between anionic nanoparticles and the negatively charged mucins, allowing for more freedom of movement within the mucus layer.^38^ This observation can be explained by the fact that anionic polymers have many surface carboxyl groups, which form strong hydrogen bonds with the mucin’s oligosaccharide chains and create bio adhesive contacts.^32^ Overall, these findings suggest that the charge of penetrating material plays a significant role in their interaction with mucins and their ability to penetrate the mucus layer in the GI tract. It may be reasonable to conclude that the barrier function of the mucus layer is small relative to the other barriers that drugs encounter during passage across the oral mucosa. This is probably a reflection of the fact that barrier function of mucus is dependent not only on the physicochemical properties of the drug but also on the physicochemical properties of the mucus.

Some regions of the oral mucosa incorporate a keratinised layer, the outermost region, comprising an orderly array of flattened hexagonal cells filled with aggregations of cytokeratin bounded by a cell envelope and surrounded by a complex mixture of lipids.^39^ This keratinised layer may be the major barrier to some drugs. Eggerth et al.^40^ investigated the in vitro transport of dextromethorphan hydrobromide and a series of short-chain alcohols and carboxylic acids across hamster cheek pouch in a Franz R diffusion cell. Full-thickness hamster cheek pouch mucosa was less permeable than tissue that had been tape-stripped, demonstrating that the keratinised layer of hamster cheek pouch was a major barrier to the transfer of the compounds studied.

Similar observations were made by Garren and Repta^41^ who studied the penetration of a series of substituted acetanilides across excised hamster cheek pouch. The permeabilities of these compounds were determined through full-thickness cheek pouch and isolated keratinised epithelial cell layer. For each compound studied the values were not significantly different indicating that the keratinised layer was acting as the major barrier to penetration for these compounds. However, additional evidence would be required to confirm that the permeability barrier was unambiguously attributable to the superficial keratinised layer. Pimlott et al.^42^ studied the absorption of prednisolone sodium phosphate across human buccal, palatal, and sublingual mucosa. Significant differences in absorption between the sites were reported which were attributed to the presence or absence of a keratinised epithelial layer which was acting as a permeability barrier. Reid et al.^43^ compared the permeability characteristics of urea and ethanol across full thickness and tape-stripped hamster cheek pouch mounted in Ussing chambers. For both these compounds tape-stripped tissue was more permeable, and the authors concluded that the keratin layer provided a significant barrier to the movement of these compounds. Based on this evidence it appears the keratinised layer, if present, presents a major barrier to drug permeability.

The morphology of the underlying non-keratinised epithelial layer presents an absorption barrier which varies according to the specific physiological site. The epithelium in the oral cavity is stratified and not tightly interconnected by junctions, but by an paracellular lipid matrix constituting a barrier for absorption. Thus, the epithelial absorption profile of drugs may vary regarding the rate and the lag time depending on both the specific mucosal epithelium targeted and the physiochemical properties of the drug. Absorption across the epithelia may occur by passive transcellular or paracellular diffusion. However, absorption may also occur because of carrier-mediated cellular uptake, as well as receptor-mediated endocytic uptake mechanisms, followed by transcytosis, which primarily takes place in monolayered mucosal epithelium. Efflux mechanisms have also been found to decrease the absorption of, primarily, small drugs across monolayer epithelia.

The paracellular transport is mainly restricted by the presence of tight junctions between the cells. This route is thus primarily for small, hydrophilic and/or charged molecules, and it is estimated to represent only 0.1% of the epithelial cell wall area.^44^ On the other hand, the transcellular route requires the passage across the lipophilic plasma membrane of the epithelial cells, and consequently it is mainly employed by lipophilic compounds, unless the DDSs aid the cellular uptake and trafficking.^10^ The uppermost 25%–30% of the epithelial layer has been proposed as the major barrier to the penetration of molecules through oral mucosa.^45–47^ Squier^48^ applied horseradish peroxidase, a water-soluble, electron-dense tracer protein with a molecular weight of 40 kDa and a size of 5–6 nm, both topically and sub-epithelially to keratinised and non-keratinised epithelium of monkey, rabbit, or rat. A biopsy was examined for peroxidase activity by electron microscopy. When applied topically, the tracer did not penetrate further than the top three cell layers. However, when the tracer was injected sub-epithelially, it penetrated through the connective tissue, the basement membrane and through the lower 75% of the paracellular spaces of the stratified epithelium. It did not however penetrate through the upper 25% of the epithelium. Further studies by Squier and Hall,^49^ revealed similar distribution patterns when tissue from various oral mucosae locations, namely gingival, buccal, labial and sublingual mucosa, ventral mucosa of the tongue and lingual fraenum, were investigated. Similar results were observed by Squier and Rooney^50^ who, with a similar experimental design, applied topically and sub-epithelially a water-soluble substance, lanthanum (2 nm in size), to keratinised and non-keratinised mucosa of rabbit or rat.

A different experimental procedure was used by Squier and Hall^51^ who incubated a 1% solution of horseradish peroxidase with small (1 mm^3^) blocks of keratinised and non-keratinised porcine mucosa. After 1 h the extent of penetration of the tracer was visualised by microscopy. The compound had a similar localisation pattern within the epithelial tissue as described previously. These results suggest that the barrier to penetration of these compounds is the same regardless of whether the tissue is keratinised or not and resides in the upper 25%–30% of the mucosal epithelium.

Further investigations were performed by Dowty et al.^52^ in which they investigated the permeability of isotopically labelled water and horseradish peroxidase across porcine gingiva, floor of mouth, and buccal mucosa in vitro. In some experiments the buccal and sublingual mucosa (both non-keratinised tissue) were tape-stripped prior to mounting. In the case of the buccal mucosa no difference in permeability was observed between the intact and tape-stripped preparations. In contrast, tape-stripped sublingual mucosa was observed to be more permeable than intact sublingual epithelium. It was concluded that in the case of the sublingual epithelium the barrier to penetration was in the superficial layers. Failure to demonstrate a difference in permeability between intact and tape-stripped buccal epithelium was attributed to the greater thickness of the superficial barrier in this tissue.

Hill and Squier^53^ used an organ culture system in which 48 explants of mucosa were maintained for periods of up to 24 days. Either lanthanum or horseradish peroxidase was placed on the epithelial surface or added to the nutrient medium around the explants. After a predetermined time, the explants were removed, and thin sections were examined by electron microscopy. At all the time periods examined the limit of penetration of these compounds was restricted to the upper one-third of the epithelium.^53^ Harvey et al.^45^ visualised the permeability barrier in the hamster cheek pouch by incubating the oral tissue with horseradish peroxidase. Examination of sections of the tissue revealed a barrier to permeability located in the superficial layers of the epithelium.^45^ Dowty et al.^52^ examined the transport of thyrotropin-releasing hormone (TRH) in rabbit buccal mucosa in vitro. Their results indicated that the upper 50 mm of epithelial tissue was a barrier to transport for this compound. Gandhi and Robinson^54^ investigated the in vitro penetration of salicylic acid through rabbit buccal mucosa. The permeability of the mucosa to salicylic acid increased in the presence of penetration enhancers. Light-microscope pictures showed that the superficial cell layer was removed after incubation with the penetration enhancer. The results suggested that the superficial layer was a major barrier to the penetration of salicylic acid.

Oral mucosa, both keratinised and non-keratinised, obtained from different regions of the oral cavities of a variety of laboratory animals has shown that a permeability barrier to the penetration of lanthanum and horseradish peroxidase is in the upper 25%–30% of the epithelial layer.^53,55^ Recent work using molecules with structures and physicochemical properties different from these tracer compounds also suggests that a superficial barrier exists in oral epithelium in the upper 25%–30% of the epithelium. This is believed to be, at least in part, as a result of the presence of transcellular lipids derived from membrane coating granules (MCG). These have a dense, central, amorphous core most likely derived from the Golgi apparatus,^56,57^ they can be found within the intermediate layers of both keratinised and non-keratinised epithelium.^58,59^ The fusing of these membrane bound lipids with the plasma membrane causes the release of lipophilic material into the paracellular spaces of the outer quarter of the epithelium.^56^ The paracellular regions of keratinised epithelium have a higher amount of nonpolar neutral lipids, such as ceramides and acylceramides. These lipids are organised in a lamellar state, contributing to the barrier function of keratinised epithelium. On the contrary, the paracellular spaces of non-keratinised epithelium have a higher amount of polar lipids, namely glycosylceramides and cholesterol sulphates. The absence of acylceramides and presence of small amounts of ceramides in the non-keratinised epithelium, as well as their amorphous state led to a higher permeability to exogenous compounds compared to the keratinised epithelium.^60,61^

The basal lamina or basement membrane has been implicated as the rate-limiting barrier to the passage of some materials or at least to offer a degree of resistance to permeants such as proteins,^62,63^ endotoxins,^64^ immune complexes,^65,66^ colloidal thorium dioxide^67^ and drugs such as chlorhexidine^68^ and beta-blocking agents.^69^ Below these layers of the epithelium lie the lamina propria and submucosa, which are composed of connective tissue and host a web of lymphatic, blood, and smooth muscle vessels. The rich vascular system underlying the oral mucosa plays a pivotal role in mucosal drug delivery.^13,26,34^ Blood vessels located beneath the oral mucosa provide direct access to systemic circulation, allowing drugs to bypass first-pass metabolism in the liver.^70^ This direct access accelerates drug absorption and onset of action, rendering the oral mucosa an attractive route for administering medications requiring rapid systemic effects.^20,26^

Finally, the immune response of the oral mucosa also plays a significant role in mucosal drug delivery, with immune cells such as macrophages, dendritic cells, and lymphocytes influencing drug absorption and distribution. Recent studies have highlighted the impact of immune responses on DDSs, emphasising the importance of understanding how immune cells within the oral mucosa can affect drug interactions and therapeutic outcomes. For example, Garofalo et al.,^71^ demonstrated that extracellular vesicles enhance the targeted delivery of immunogenic oncolytic adenovirus and paclitaxel in immunocompetent mice. Studies such as these showcase the potential of immune-mediated drug delivery strategies. Additionally, Golshani et al. discussed recent advances in oral mucoadhesive drug delivery, emphasising the role of immune responses in delivering biological drugs effectively, such as antimicrobial peptides.^72^ These studies underscore the intricate interplay between the immune system and mucosal drug delivery, highlighting the need to consider immune responses in optimising drug delivery platforms for enhanced therapeutic efficacy.^71,72^

## Mimicking the mucous membrane using in vitro experiments

Mimicking the mucous membrane using in vitro experiments is a crucial aspect of drug testing platforms, particularly in the development of oral mucosal models. Recent studies have advanced our understanding of oral mucosal drug absorption kinetics and bioavailability through innovative DDSs, nanocarriers, and mucus-penetrating technologies. The clinical pharmacokinetics of drugs administered via the oral mucosa are influenced by factors such as water content, bioavailability, and the properties of the mucous membrane itself. Understanding how drugs interact with the oral mucosa and are absorbed into systemic circulation is crucial for optimising drug dosing regimens and enhancing therapeutic outcomes. By elucidating the absorption mechanisms and pharmacokinetic profiles of drugs delivered through the oral mucosa, researchers can tailor drug formulations to improve efficacy and minimise side effects.^73^

Over the years, oral mucoadhesive films have gained prominence as effective mucosal DDSs due to their unique characteristics such as ease of administration, rapid onset of action, and high bioavailability.^74^ These films provide a platform for localised drug delivery to the oral mucosa, offering a promising approach for treating various diseases of the oral mucosa. Combining biofilms with immune-response modifiers has been explored as a strategy to enhance drug delivery to the oral mucosa, indicating the potential of integrating different technologies to address challenges associated with oral mucosal drug delivery.^75^

Salivary mucin molecules and their negative charge play significant role in facilitating drug delivery through the oral cavity. Salivary mucins coat the oral cavity and can interact with positively charged drug molecules, aiding in their delivery to specific tissues. This interaction is particularly useful in the development of mucoadhesive systems, where the goal is to enhance the retention of drugs at mucosal surfaces. Researchers utilise models involving mucin-polymer interfaces to understand the mechanisms underlying mucoadhesion. The adhesive strength observed in mucoadhesive systems is attributed to molecular bridges formed between mucin and polymers. Additionally, the electrostatic properties of mucin contribute to mucoadhesion, further enhancing the interaction between mucin and polymers.^72^

Alqahtani et al.^76^ discussed the use of polymeric nanocarriers to deliver insoluble drugs, target drugs to specific regions of the GI tract and facilitate drug transcytosis across mucosal membranes. Liu et al.^77^ focussed on mucus-adhesive nanoparticles for oral drug delivery, emphasising the importance of nanoparticles that allow for prolonged contact between drugs and mucosal membranes to enhance drug delivery efficiency. Boegh and Nielsen^78^ explored the barrier properties of mucus and its impact on drug delivery, highlighting the need to address mucus as a critical barrier for achieving sufficient bioavailability of orally administered drugs. Stewart et al.^79^ investigated the impact of drug-rich colloids on membrane flux and oral bioavailability, suggesting that designing amorphous formulations producing colloids upon dissolution could improve drug bioavailability for compounds with low solubility and high permeability. He and Mu^80^ discussed microenvironmental pH modification in buccal/sublingual dosage forms to optimise drug absorption at the oral mucosa, balancing drug solubility and permeation for effective drug delivery. Sato et al.^81^ highlighted the role of mucopenetrating and mucoadhesive nanocarriers in rapidly delivering drugs to absorption sites and prolonging residence time near the absorption membrane, enhancing medication efficacy.

A frequently used method to predict the adhesive properties of such DDSs is utilisation of a mucin adsorption assay in which the PAS staining technique is applied as a measure of mucin adsorption to material surfaces. This assay provides a colourimetric reading which can be used to calculate the mucin binding efficiency of the material in question.^82–85^ Whilst many studies to date have used this method of predicting the mucoadhesive properties of materials, and it proves effective in analysing the level of mucin interaction with polymers, the method does not provide the full picture of the efficacy of drug diffusion through the mucin layer. For this, much more sophisticated analyses are required.

In this realm of membrane mimetics, phospholipid bilayer nanodiscs have been utilised to characterise integral membrane proteins like the voltage-dependent anion channel (VDAC-1), providing insights into the structural and functional properties of these proteins within lipid bilayers. This approach offers a way to mimic the cellular membrane environment and study the behaviour of membrane proteins, which could be valuable in understanding drug interactions with membrane-bound targets in the context of oral mucosal drug delivery.^86^

Several strategies for mimicking the mucous membrane in vitro have been described. One option is the isolation of native mucus, often obtained from the female genital tract or from cystic fibrosis patients. However, as with all naturally derived materials, attention should be given to the batch variation and differences in properties between different sources.^87,88^ Animal mucus can also be obtained in reasonable quantities and used with or without further purification. Alternatively, mucins can be extracted from native mucus and used to form gels.^27^ Mucin products are also available commercially, however because of purification and processing of these mucins it is impossible to obtain rheological properties which resemble that of native mucus. It is proposed that the mixing of mucus from different sources could possibly tailor properties to better mimic the native conditions. Implementation of such methods would require rigorous standardisation, characterisation and testing to ensure reproducibility and relevance to the intended application. In terms of setting up these sorts of acellular models, usually researchers use a mucus application over a filter insert in a transwell plate insert to create donor and recipient chambers for analysis.^89,90^

Another possibility is the use of mucus-secreting cell lines, these are typically used to produce models of mucus covered epithelia, but it could be an option to harvest the mucus from cells and use this as a model itself. Quantities may be low and methods would require significant upscaling but this is an alternative which could help in overcoming some ethics concerns. Cell lines derived from colonic (HT29 and LS174T) and bronchial (Calu-3) carcinoma have shown the ability to differentiate into mucus-secreting cells.^91–93^

The use of cell culture models which incorporate an artificial mucus layer have also been described due to increased interest in the on mucus as a barrier to drug delivery. The production of a biosimilar artificial mucus has been described by a number of groups and generally involves the production of a solution containing a mix of polyacrylic acid, mucin, bovine serum albumin, polysorbate and lipids such as cholesterol, linoleic acid and phosphatidylcholine.^88,94^ Studies by Birch et al.^95^ have shown that the artificial mucus doesn’t damage the integrity of epithelial cells or impact cell viability. These methods have been applied specifically to a buccal in vitro model, this model was based on an adherent freeze-dried mucus layer deposited onto the TR146 epithelial cell line and was proved effective in assessing the transmucosal transport if nanomaterials.^96^ Boegh et al. have investigated a model using Caco-2 cells, matured for 18 days on a filter insert followed by covering with biosimilar mucus. This model showed that the biosimilar mucus formed a barrier to both lipophilic and hydrophilic drugs, with the most profound effect seen on lipophilic compounds as is consistent with other data on the effects of the mucus layer on drug diffusion.^88^

These findings provide valuable insights for designing effective drug delivery platforms targeting the oral mucosa and improving therapeutic outcomes for various mucosal conditions.

## The role of saliva in oral drug delivery

The roles of saliva in oral mucosal drug delivery are multifaceted and pivotal in optimising therapeutic outcomes. Saliva acts as a crucial medium influencing drug delivery through various mechanisms. Saliva aids in drug absorption through passive transport, utilising transcellular and paracellular pathways, with the choice of route dependent on the drug molecule’s characteristics.^80^ Saliva is rich in proteins, electrolytes, and enzymes and plays a significant role in drug dissolution, release, and absorption in the oral cavity.^97^ Saliva’s lubricating function and unique protein milieu promotes wound healing, cell migration, and antimicrobial activity, contributing to a healthy oral microflora and facilitating drug delivery.^23^ Saliva also provides a water-rich environment that aids in drug dissolution and release from buccal and sublingual formulations, enhancing drug permeation through the oral mucosa.^80^

Changes in the pH level of saliva can affect the way drugs are absorbed in the body. The degree of ionisation of a drug is influenced by the pH of the surrounding environment. When drugs are taken, they can passively absorb through either transcellular diffusion or paracellular diffusion, depending on their physicochemical properties. The most prevalent method, transcellular diffusion, is more efficient for drugs that are in a non-ionised state because they are more soluble in lipids. Therefore, drugs with higher pKa values, indicating a tendency to remain non-ionised, are preferred for absorption in areas like sublingual and buccal where saliva has a neutral pH. On the other hand, drugs that are hydrophilic or ionised are better absorbed through the paracellular pathway. It’s important to note that the pH of saliva can change temporarily due to factors like food and drinks or oral health issues, which can affect how drugs are absorbed when administered in sublingual and buccal routes.^11,34^

The continuous flow of saliva and swallowing actions in the oral cavity can impact drug residence time on the oral mucosa, influencing therapeutic efficacy.^98,99^ The rate at which the drug formulation breaks down and the drug dissolves can be influenced by the amount of saliva present. For instance, if the mouth is dry, this can hinder the absorption of the drug. On the other hand, excessive saliva flow can cause the drug to be swallowed before it has a chance to be absorbed through the oral mucosa.^34^ Saliva is composed of >99% water, its pH and composition including its constituents, are influenced by the rate of saliva flow. The flow rate of saliva is highly variable and can also be affected by food intake. Increased saliva production, often stimulated by food consumption can wash away hydrophilic drugs from their site of application shortening the drug’s retention period in the oral cavity. It continuously bathes the oral mucosa, dilutes the drugs, and can lower the absorption and bioavailability of a topically administered medication and ultimately impact its therapeutic effectiveness. This phenomenon is called the ‘salivary washing effect’. However, it remains unclear whether the salivary secretions influence the diffusion of the deposited drug deeper into the tissue.^12,18,22,26,100^

Several factors such as age, medications (such as anticholinergic drugs), and medical conditions (such as Sjögren’s syndrome, cheilosis, glossodynia, dehydration, dysphagia, and mastication problems) can affect saliva flow, thereby impacting the effectiveness of buccal and sublingual drug delivery.^34^

Serpe et al. conducted an in vitro evaluation study of salivary washout on drug delivery to the oral cavity using sulforhodamine (SRD)-coated microneedles. They found that salivary flow, both dynamic and static, increases drug penetration, which modifies the kinetics of permeation. There is also a considerable drug backwash caused by presence of a dynamic salivary flow, with 90% of the SRD lost into the donor chamber’s PBS. On the other hand, although the loss increased from 14% to 37% when the static volume of PBS in the donor chamber was increased from 100 to 300 μL, this was a much less significant impact than the dynamic flow instance. This evidence implies that, in order to minimise drug loss via salivary washout, it may still be necessary to shield the area of insertion with a protective mucoadhesive covering or patch.^12^

The described washing effect of saliva and mechanical stress promote the physiological removal of drugs from the oral cavity and take the formulation away from the mucosa, resulting in a relatively short exposure duration and variable drug distribution at the area of deposition. Therefore, therapeutic drug levels in the mucosa and circulation cannot be guaranteed by standard dosage forms for mucosal and transmucosal delivery.^101^

## Effectiveness and feasibility of oral mucosa modelling for validating drug delivery

When designing preclinical in vitro models, the feasibility and accessibility are important factors to consider. There are several areas where these considerations should be made to ensure the production of a reliable reproducible model, which will be discussed throughout this review. Briefly, the cell source should be carefully selected so as it is readily available, scalable, standardisable, and comparable to models used in previous studies.^102^ Additionally, any methods, culture vessels or other materials used in the model development need to be easily adoptable and standardisable. Many of the methods of oral mucosal model development presented throughout the literature describe the use of scaffolding materials which are fabricated in-house requiring complex assembly or customised machines for production, making adoption of the model in drug delivery studies by other groups more difficult.

The development of engineered tissue models should aim to augment and complement existing drug development models throughout the validation process. For example, existing animal models are time consuming, low throughput, difficult to analyse and poor representatives of in vivo human tissues. Therefore, here the development of complex in vitro tissue models shows promise in the advanced screening of drugs to progress to animal/clinical trial stages. However, as the biological relevance of in vitro models comes into question the models begin to become increasingly sophisticated, thus coupled with a decrease in the ease of model assembly and throughput.^103^

In relation to the feasibility of using these models in drug development one should also consider the propensity for data collection from assays readily conducted in drug discovery investigations. Typically, the data output potential decreases as the complexity of a model increases, it cannot be assumed that assays developed for use with monolayer cultures are suitable for direct translation for use for 3D tissue models. A key consideration which should be made in this area is the size of molecules required for the completion of the assay and the barriers to penetration of these molecules through the 3D layers of cells for interaction with the central components of the model.^104^ Some assays rely on cell lysis for functionality and penetration of reagents is necessary to achieve uniform cell lysis of all components of the model. Another example may be in relation to the diffusion of fluorescent probes and the ability for photons to penetrate the tissue model for probe excitation or fluorescent emission and the effects this may have on the ability for fluorescent imaging of the samples. Whilst optical assessment techniques which have previously been widely applied in 2D culture models have been adapted for use with 3D culture they are typically endpoint or static measurements which are time inefficient for large-scale analysis.^105^ Whilst this is a significant barrier to the widescale use of complex in vitro tissue models for drug delivery applications it has been shown that some traditional screening methods can be validated for use with 3D tissue models on a case-by-case basis.^106,107^ Additionally, interest in the modification of electrical monitoring techniques for the analysis of complex cultures has been described.^108^ These methods of electrical sensing may allow the dynamic, real-time, and label-free monitoring of cells in 3D cell culture models, thus overcoming some of the challenges associated with analysing cell responses in these types of models.

Taking the above into account it becomes clear that there is a balance to be struck between the model complexity, availability, and reproducibility in addition to other design considerations such as the predictive capacity or biological relevance of the model. This needs to be assessed on a case-by-case basis and is dependent on the disease under investigation or the method of drug delivery to be employed in the investigated system. A significant barrier to the development of biologically relevant and complex models appears to be obtaining suitable experimental data against which the model can be validated. Ultimately, for the use a preclinical in vitro model to become successful in the evaluation of drug delivery characteristics the results should be compared to the preexisting body of literature.^109^ However, unless a model begins to become widely adopted this is difficult to achieve.

## Considerations for in vitro tissue models

**Figure 2.**
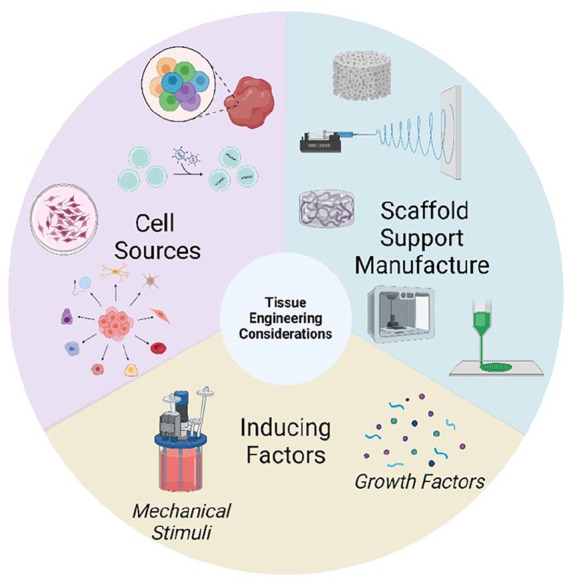
General considerations which must be made when developing tissue engineered in vitro models for use as in vitro test systems in the investigation of drug permeability and efficacy of new DDSs.

The classic description of tissue engineering involves the combination of biomaterial scaffold support, living cells and physicochemical stimuli. The combination of these features should aid in the development of a biological substitute which mimics as wells as possible the histological structure, mechanical properties and functionality of the tissue condition being modelled. The field of tissue engineering therefore combines the fields of cell biology, materials science and bioengineering to design these complex in vitro models. Due to the unique nature of each tissue, the development of in vitro models becomes a very complex process which requires consideration of the following factors which will be discussed at length in this section: biomaterial fabrication techniques, biomaterial sources, cell sources and chemical/physical stimuli as summarised in Figure 2.

### Biofabrication methods for 3D tissue models

Among considerations for designing an appropriate model system should be the selection of an adequate processing or fabrication method of biomaterials. This technique may be either conventional or advanced manufacturing and should aim to reduce the fabrication time and enhance the reproducibility of the final model. One of the more traditional methods of scaffold fabrication involves solvent casting particulate leaching to create highly porous matrices which have been applied to hard tissues. Typically, this technique involves the dispersion of a salt which is insoluble in a polymer solution until a homogenous dispersion is achieved. This composite solution is then solvent cast and allowed to dry via solvent evaporation, the resulting matrix is then submerged in water to facilitate leaching of the salt from the matrix. These scaffolds have been reported to yield scaffolds of up to 90% porosity with good pore interconnectivity.^110^ A key advantage of these highly tuneable porous structures is the ability for cells to migrate throughout the scaffold architecture.^111^ Similar scaffold architectures have been achieved using techniques such as freeze-drying, gas foaming and thermal-induced phase separation.^112–115^

More recently 3D-bioprinting (3DP) has become popular for the production of scaffolds during in vitro modelling and tissue engineering practices, a number of common 3DP technologies are frequently discussed, extrusion-based printing, inkjet printing, laser induced printing and stereolithography. Typically, these techniques involve the delivery of a cell laden biomaterial (bio-ink) to a build platform, the bio-ink is extruded from a needle and can be patterned to a reflect a design generated in CAD. The model is then built up in a layer-by-layer fashion from bottom to top requiring a steady material flow and a material which rapidly stabilises after deposition. Examples of materials frequently used in this technique include alginate and gelatin-methacrylate (GelMA) which demonstrate rapid stabilisation via interaction with calcium ions or cooling/photo-induced cross-linking respectively. GelMA has been used both alone and in combination with other polymers to produce 3DP constructs with optimised properties for a range of in vitro modelling applications.^116–118^ Alginates have often been used in combination with other materials used in 3DP technology. Whilst alginates have many favourable properties such as gelling, viscosifying and stabilising characteristics alongside good biodegradability and biocompatibility, the printability of this polymer is poor and therefore modifications using other polymers are often described.^119,120^ 3DP offers excellent versatility as many controllable parameters such as bio-ink composition, printing speed, extrusion pressure, scaffold geometry and needle diameter allow complete tailor ability of the scaffolds produced. Whilst many advantages are experienced using this technique there are some limitations to the use of 3DP for tissue engineering applications. Despite the wide range of available biomaterials, not many of these demonstrate the gelling properties required to stabilise the final construct. Additionally, some improvements on print resolution are required to achieve intricate geometries and the encapsulation density of cells in bio-inks remains challenging.^121^

Electrospinning is another technique which has been described to produce scaffolds to be used for in vitro modelling purposes. Electrospinning relies on the projection of a fine polymer jet via the induction of an electric field between a charged needle tip and grounded or oppositely charged collector. Typically, a polymer solution or melt is used for the formation of the fine polymer jet, to successfully establish a stable jet the electrostatic repulsion induced by an applied high voltage must overcome the surface tension of the polymer liquid until a critical point is reached and the ‘Taylor cone’ is formed.^122^ Electrospinning produces non-woven mats of ultra-fine polymer fibres in the nano-micrometre range. In the past, cells have been subsequently delivered to the scaffolds following fabrication using cells suspended in culture media.^123^ However, more recently progress has been made on incorporating cells within electrospinning solutions to generate in-situ cell-laden fibrous scaffolds.^124,125^ This is an inexpensive technique which offers the possibility to tailor material properties via control of flow rate, applied voltage, solution viscosity and collector distance, however limitations such as inhomogeneity of cell dispersion and difficulty in achieving 3D architectures have been described.^125,126^

### Biomaterials

In vivo, cells reside within a matrix composed of proteins, glycosaminoglycans (GAG) and glycoconjugate known as the extracellular matrix (ECM). In nature this provides a physical scaffold, mechanical stability, and biochemical cues to maintain homoeostasis and support morphogenesis. In vitro modelling of tissues requires such a scaffold to mimic the native tissue matrix for cellular support. Choosing a biomaterial for this scaffold is of critical importance and depends on the tissue which is being modelled. There are three main categories of scaffolds: polymers, ceramics and metals. There is also the potential to combine these to form composite materials.^127^ The selected biomaterial should provide adequate support for cell attachment, proliferation, and migration; additional consideration should be given to the degradability and mechanical properties of the material. Materials should be at minimum biocompatible, if not bioactive via incorporation of biological cues and growth factors in a tissue specific manner. Both natural and synthetic polymers have been described for use in in vitro tissue modelling, a summary of previously investigated polymers, metals, and ceramics for use in such application is provided in Table 1. By enlarge the presented evidence shows that whilst natural biomaterials offer enhanced biological properties, in most cases due to the presence of biologic cues, often the mechanical properties are compromised. The opposite is true for synthetic polymers and therefore often researchers employ the use of natural-synthetic polymer blends to tailor the properties for the specific application.

**Table 1.** Polymers which have been reported for use when in vitro modelling tissues.

Biomaterial	Category	Advantages	Limitations	Ref.
Chitosan	Natural Polymer	Biocompatible, mucoadhesive, biodegradable, inherent antibacterial properties and similar structure to GAGs.	Comparatively poor mechanical properties.	Ahmed et al.^128^
Hyaluronic Acid (HA)	Natural Polymer	Biocompatible, biodegradable, good cell viability/proliferation and good printability.	Ethical concerns with the use of animal derived HA, scalability problems with large-scale production for commercial use.	Gallo et al.^129^
Gelatin	Natural Polymer	Inexpensive, easily modifiable, biocompatible, biodegradable.	Poor mechanical properties, brittle, very rapid degradation.	Lukin et al.^130^
Collagen	Natural Polymer	Biodegradable, biocompatible, highly versatile and easily isolated from a range of sources.	Difficult to sterilise without altering structural changes.	Muthukumar et al.,^131^ Parenteau-Bareil et al.^132^
Fibrin	Natural Polymer	Readily interacts with platelets, leucocytes, fibroblasts, and endothelial cells. Promotes cell migration and tissue ingrowth.	Rapid degradation, poorly understood shrinkage behaviour and poor mechanical properties.	Sanz-Horta et al.,^133^ Noori et al.^134^
Alginate	Natural Polymer	Biocompatible, biodegradable, non-toxic, chelating properties, rapid gelation and hygroscopicity.	Poor mechanical properties, low solubility and unsuitable degradation.	Farshidfar et al.^135^
Silk Fibroin	Natural Polymer	Biocompatible bioactivity, good mechanical strength, sustainable material, and biodegradability.	Complex processing required, poor gelation properties and low moulding ability.	Lujerdean et al.,^136^ Liu et al.^137^
Decellularised ECM	Natural Polymer	Readily available, abundant bioactive cues, provokes relatively little immune response and retains vascular networks.	Batch to batch variability, expensive and animal derivation introduces problems with cross species disease transfer.	Londono and Badylak^138^
Polycaprolactone	Synthetic Polymer	Biocompatible and moderate mechanical strength.	Slow degradation, poor cell adhesion, hydrophobic and inflammatory responses.	Malikmammadov et al.^139^
Polylactic Acid	Synthetic Polymer	Biocompatible and biodegradable.	Brittle, low cell adhesion and inflammatory responses.	DeStefano et al.^140^
Poly(ethylene glyol) (PEG)	Synthetic Polymer	Biocompatible, biodegradable and can be easily functionalised.	Moderate mechanical strength, low cell adhesion, problems with scalability, poor printability and reports of anti-PEG antibodies due to frequent use in pharma industry.	Zhang et al.^141^
Poly Lactic-co-Glycolic Acid	Synthetic Polymer	Good biocompatibility and adjustable biodegradability.	Poor cell affinity and acidic byproducts	Jin et al.^142^
Polyurethanes	Synthetic Polymer	Tailorable mechanical properties, bio-adhesive properties	Poor degradability and requirements for co-polymerisation.	Alves et al.^143^
Hydroxyapatite	Natural/Synthetic Ceramic	Bioactive, biocompatible, osteoconductive and hydrophilic.	Brittle, low tensile strength, and low fracture toughness.	Ghiasi et al.^144^
Ceramics	Synthetic	Osteoinductive, osteoconductive, low toxicity, potential to induce angiogenic response and potential for sustainable manufacturing.	High brittleness, poor mechanical properties for load bearing applications.	Punj et al.^145^
Metals	Synthetic	Good mechanical properties and low degradability.	Ion release may cause cytotoxic responses and subject to oxidation.	Radenković and Petković^146^

### Cell sources

With respect to cell sources, it can be difficult to find the most appropriate cell source for the tissue-engineered models. In some cases, this can be dependent on the availability of tissue-specific cellular phenotypes providing the capability of representing the characteristics of normal or disease state tissues. Additionally, the density of cells which can replicate the in vivo tissue should be carefully considered. As the key benefit of developing these in vitro tissue models is to close the gap between animal models and clinical trials it is most appropriate to use human cells in the model development.

Most of the in vitro models currently described utilise adult primary cells which have been isolated from patients. These cells are isolated from tissue biopsies, healthy or diseased, and therefore represent well the functional in vivo tissue; however, some problems are presented when using these primary cell sources. Firstly, these cells have a limited lifespan and demonstrate slow proliferation rates. Additionally, the isolation procedures can be complex and there is potential for contamination with unwanted cell types.

To overcome issues associated with primary cell isolation one may opt for the use of immortalised cell lines due to ease of access, expandability, and reproducibility. Many models have been developed using immortalised cell lines however, the behaviour of these is not always similar to cells harvested from in vivo biopsies.^147,148^ Elsewhere, Buskermolen et al.^149^ describe the limitations associated with the use of primary cells for development of in vitro models due to limited availability of biopsy donors. Their study compared the use of immortalised cell lines in the development of a model to their primary cell equivalents, showing similarities between the two models produced. Therefore, it is concluded that the similarities of immortalised cell lines to primary cell equivalents for use in the production of in vitro tissue models should be assessed on a case-by-case basis.

To overcome limitations with both primary cell cultures and immortalised cell lines for in vitro modelling, stem cells have been investigated in some areas.^150,151^ These are undifferentiated cells which can be isolated from a range of different sources: embryos, foetuses, and adult tissues such as bone marrow and other stem cell niches are all sources of stem cells. Stem cells can self-renew and differentiate into numerous cell types, with the differentiation potential being dependent on the original stem cell source and subsequent environmental stimuli encountered. The key limitations of using stem cells are the ability to control the differentiation pathways towards the desired lineage and the fact that differentiated stem cells often display immature phenotype with gene expressions equivalent to that found in foetal cells.^152,153^ Additionally, induced pluripotent stem cells (iPSC) have been engineered from differentiated somatic cells via the induced over expression of specific transcription factors.^154^ Since their first description in 2006 these iPSCs have been used in the in vitro tissue modelling of disease state via isolation of cells from patients with a specific pathology thus allowing the modelling of the disease.^155^

### Culture conditions

In vivo environments ensure the presence of molecular and mechanical cues which direct cell behaviour. These stimuli can influence factors such as mitosis, cell shape, cellular spreading and proliferation and secretion of ECM components. Additionally, in vivo, the presence of a vascular system throughout tissue ensures the adequate provision of nutrient supply and waste removal. During the design of in vitro models, it should be considered that cells in the centre of the organoid may be behaving differently to superficial cells depending on the ability of nutrients to reach the centre of the construct. Often the prevention of successful in vitro model development has been caused by limited nutrient and waste diffusion. To avoid this limitation mechanisms such as mechanical and chemical signals can be used. These stimuli were traditionally induced using bioreactors designed to reproduce the in vivo growth conditions, however, recently novel platforms based on microfluidics have become important tools which can be used for this purpose.^156^ Using microfluidics in this application has shown promise due to an excellent potential to reproduce sophisticated in vitro organ models, such as the skin. The technology employed here incorporated an automated and biomimetic system to better simulate the dynamic environment encountered in vivo. Microfluidic systems may incorporate in situ biosensors for non-invasive testing which can further aid in the realm of drug discovery and testing.^157^

An additional consideration is the selection of culture media for co-culture conditions. The culture media is used to nourish cells usually consisting of the base medium, serum and regulating factors. The specificities in these compositions are important as they determine cell fate and differ for each cell type. Establishing an appropriate medium when two or more cell types are present becomes challenging. Several approaches have been described in attempts to overcome these limitations. Mixed medium is the most simplistic of the methods here and involves mixing the culture media for all cell types present in an appropriate blend ratio.^158^ It should be considered that the supplements present within some media may interfere with other cell types within the co-culture. Another approach is to use a very general base medium and supplement it with soluble factors which can stimulate one cell type without negatively effecting the others, offering more specific modulation of the media than just mixing two complete medium compositions. Unfortunately, it is time consuming and difficult to find suitable supplements to optimise the combined media. Finally, a culture system enabling two partitioned media flows can be used, meaning that each cell type in a co-culture can receive their respective media whilst cell-cell contact is maintained.^159^ These systems do have limitations and tend to only work for 2D cultures or specific cell types.

## In vitro modelling techniques

Given the increased effort in enhancing the efficacy of drug delivery and bioavailability of drugs in recent years an extensive range of in vitro modes have been reported. This includes several models of the oral mucosa. Designed models aim to mimic as closely as possible the native tissue environment in which the DDS will be administered, therefore each complex in vitro model should be tailored to the specific final application. This may introduce complexities within models for testing of DDSs which are for local treatment of diseases, for example infections or trauma injuries to the oral mucosa. In cases such as these the in vitro model should be designed to incorporate characteristics of the disease/injury state. Whereas, for the testing of a DDS designed for systemic delivery via an oral transmucosal mechanism an in vitro model of the healthy oral mucosa may be appropriate. Since 1995 researchers have been working to develop in vitro models of the oral tissues. In this year a group produced an in vitro oral epithelial model to investigate cell permeability to adrenoreceptor antagonists.^160^ This was achieved using TR146 human cells, a cell line derived from a human buccal carcinoma, which to this day remains one of the most popular cell lines for use in this application.^161^ A commercial model is available using this cell line, the human oral epithelium produced by SkinEthic Laboratories (Nice, France) suggesting that this is a reliable cell line for creating reproducible models of oral epithelium. Models have been designed to cover investigation of healthy, ulcerative, fungal and bacterial infection state oral mucosa. Within the following section the development of such models will be discussed.

### Healthy oral mucosa

An in vitro model of the oral mucosa with permeability characteristics comparable to normal oral mucosa was first described by Selvaratnam et al.^162^ Keratinocytes obtained from several sources; buccal mucosa, hard palate and abdominal skin were cultured on a commercial collagen membrane (Cellagen^®^) or on dead decellularized dermal tissue. These cultures were initially grown in submerged conditions before exposure to the air-liquid interface. By enlarge the keratinocytes grown on the decellularized tissue displayed morphology closer to that of native tissue presenting with a thicker epithelial layer, ordered stratification and a polarised basal layer with good attachment to the substrate. The oral models investigated displayed water permeability characteristics similar to that of the corresponding native tissues (buccal and hard palate). Analysis of lipid production showed that all models contained the major lipid groups usually found in epithelial tissues. Despite the presence of phospholipids being significantly decreased for the in vitro model tissues other lipid groups and sterols such as cholesterol, glucosylceramides and ceramides were comparable to native tissues in most cases. Given the similarities in permeability properties of the models described by this group they could serve as good in vitro drug testing platforms for the oral mucosa.

In 2003 Costea et al.^163^ described the necessity of fibroblasts and keratinocyte growth factor (KGF) to produce oral mucosal models with morphological similarities to in vivo tissue. Results showed that whilst models without fibroblasts were able to stratify in a monoculture of keratinocytes on a collagen matrix, the resulting model epithelia were thin with loose attachment to the collagen matrix in comparison to native epithelia. Co-culturing the keratinocytes atop a fibroblast embedded collagen matrix significantly increased the epithelium thickness from 28 to 66.1 µm. The effect of KGF appeared to increase the model epithelial thickness regardless of the presence of fibroblasts. This response was dose dependent, with the mucosal thickness increasing as the concentration of KGF increased. This trend was also observed with the fibroblast embedded models where the co-culture system incorporating 10 ng/ml KGF bearing the greatest similarity in overall thickness to native mucosal epithelium. Additionally, this model performed closest to the native oral epithelium in the other aspects such as proliferation and apoptotic indices in both the basal and suprabasal cell layers. It was concluded that the inclusion of fibroblasts in the model had a more profound effect on the proliferation and differentiation of in vitro model oral mucosa than the inclusion of KGF, therefore this model was used in further studies for pharmacological applications.^164^ The models were used to assess the effect of glycerol, which is often used as a treatment for dry mouth in clinical settings, on epithelial homoeostasis and tissue integrity. Matured oral epithelial models were exposed to different concentrations of glycerol (17%, 42.5% and 85%) and analysed using immunohistochemistry, H&E staining and Ki-67 staining. Results showed that the high concentrations of glycerol > 42.5% caused increased epithelial cell proliferation, thickness and apoptosis compared to controls treated with water only. E-cadherin staining showed no significant changes in the tissue integrity following treatment of the tissue engineered oral mucosae. This research showed the successful application of the in vitro oral mucosal models to pharmaceutical testing, consolidating the usefulness of these models for drug discovery and validation.

An early comprehensive investigation of 10 natural/synthetic biomaterial scaffolds in conjunction with a co-culture of fibroblasts and keratinocytes isolated from biopsies was performed by Moharamzadeh et al.^165^ Studies performed by the group highlight the importance of biocompatibility, biostability and porosity of the scaffold material selected to successfully mimic the oral mucosa for in vitro testing applications. Pore size drastically effected the fibroblast infiltration and interaction with keratinocytes as models utilising commercial collagen and collagen/elastin materials with poor porosity demonstrated little to no interaction between the two cell types. Whilst porosity was an important factor this should be closely controlled as highly porous scaffold morphologies resulted in keratinocyte invasion leading to the formation of epithelial islands throughout the scaffold structure. Therefore, lamination of non-commercial synthetic scaffolds using Matrigel^®^ improved the formation of separated, well-developed epithelial layers. Additionally, the importance of exposure of the construct to the air-liquid interface is highlighted as a driver of epithelial differentiation. In this study the biomaterial which best supported the development of a multi-layered stratified epithelium was a freeze-dried collagen-GAG scaffold with Matrigel^®^ lamination prior to keratinocyte seeding. The co-culture system combined with this biomaterial supported fibroblasts within the scaffold spaces capable of producing connective tissue components. Additionally, this in vitro model supported the differentiation of the TR146 keratinocyte cell line to form a non-keratinised superficial epithelial layer.

In other 3-D tissue models researchers have utilised commercially available biomaterials such as collagen-elastin matrix (Matriderm^®^) alongside co-culture systems of fibroblasts and keratinocytes for the development of an in vitro oral mucosal model.^166^ Fibroblasts were allowed to infill the Matriderm^®^ matrix and mature for 14 days in vitro before seeding of keratinocytes atop the developed fibroblast layers. After a total of 3 weeks culturing of the model in submersed conditions the model was lifted to the air-liquid interface and cultured in media supplemented with ascorbate-2-phosphate (A2P) and human keratinocyte growth supplement (HKGS) for 10 days. The model successfully supported the growth and differentiation of the gingival fibroblasts and keratinocytes for the duration of the study. At 2 weeks post-seeding with the fibroblasts, they had successfully infiltrated and covered the Matriderm^®^ forming a dermal area. This surface was well receptive to the settling and development of a keratinocyte layer as the large pores in the scaffold had become infilled with fibroblasts. Following the exposure of the construct to the air-liquid interface with modified media for 10 days, the development of a continuous epithelial layer was formed, suggestive of the potential for a functional barrier epithelium in the completed model. Despite Moharamzadeh et al.^165^ reporting the lack of suitability of their collagen-elastin matrix for this application, this more recent study demonstrated that by altering the morphological properties of the scaffold the material can be suitable for the support/development of a mucosal model. The reports here support the investigations by Lin et al.^148^ showing the advantages of exposure to air and selected growth supplements in forming a barrier membrane.

To try to provide some standardisation to the production of oral mucosal equivalents, Jennings et al. investigated the use of a commercial TERT2-immortalised oral keratinocyte cell line (FNB6) as an alternative to using primary normal oral keratinocytes (NOK).^167^ The results showed that this alternative could provide the potential for much more standardised protocols in research within this field. The substitution of NOK for FNB6 did not significantly impact the characteristics of the engineered mucosa when compared to human oral mucosa or the NOK derived mucosal equivalent. Additionally, similar trends in cytokine expression (CXCL8 and ICAM-1) are obtained across the three groups when stimulated using IL-1β, TNFα and/or lipopolysaccharide. Recently, this oral mucosal model has been successfully applied for the in vitro analysis of drug delivery through mucosal tissue.^168^ The effects of this drug delivery via a polymer patch were analysed via cytokine expression changes and the tissue model successfully acted as a testing platform for novel DDSs as an alternative to in vivo models.

More recently a group reported for the first time the development of a full thickness 3-D tissue engineered model encompassing both the mucosal and alveolar bone components of the oral anatomy.^169^ Establishment of this model involved the combination of two different scaffold structures which were cultured separately and then combined using a biocompatible fibrin sealant. The bone model was supported using porous ceramic hydroxyapatite/tricalcium phosphate discs, the model was maintained in a spinner bioreactor using a rat osteosarcoma-derived cell line as the cell source. For the development of the mucosal portion of the model a freeze-dried collagen scaffold was used in combination with a co-culture of primary fibroblasts and immortalised human oral epithelial cells (OKF6-TERET-2). Following combination of the constructs the model was further cultured at the air-liquid interface for 5 days to induce differentiation. The model histologically represented the in vivo alveolar bone-oral mucosa complex. In 2018 the group provided an update to the model using only cells isolated directly from oral tissues.^170^ Primary human oral keratinocytes and oral fibroblasts were isolated from the gingival tissue whilst primary alveolar osteoblasts were isolated from bone chips collected during preparation of dental implant sites. This model underwent more rigorous testing than the initial model in 2016 and via q-PCR it is proven that the model expressed similar levels of epithelial differentiation markers as native tissue. Additionally, analysis of osteoblastic markers showed that throughout the 2-month culture period the human osteoblasts in this model maintained normal phenotypic characteristics.

Lin et al.^148^ describe the optimisation of a model system to increase the paracellular barrier of in vitro mucosal models. This is an extremely important factor in utilising in vitro models for the investigation of drug delivery studies. This model used the TR146 cell line in a range of different culture conditions to optimise the development of a clinically relevant model with barrier functions comparable to native mucosal tissue. Using the air-lift cultivation technique during these studies resulted in increased transepithelial electrical resistance (TEER) values, especially when cultivated using EpiLife media as opposed to DMEM. Following further investigations using supplementation of DMEM with factors such as hydrocortisone, human keratinocyte growth supplement (HKGS), KGF, A2P and foetal calf serum (FCS) under both submerged and air-lift culture conditions it was concluded that the most representative barrier function was obtained using DMEM supplemented with 1% HKGS and 10% FCS under air-lift conditions. HKGS contains EGF and hydrocortisone which have been shown to enhance differentiation of epidermal barriers and to promote tightness of neural endothelial cells respectively.^171–173^ The optimal culture conditions described here not only proved to increase the barrier membrane function via TEERs investigation, additionally a high-throughput qPCR investigation confirmed the expression of several tight junction markers in the optimised model, showing similar expression to samples extracted from biopsies of oral mucosa. Additionally, cornification markers, loricrin, filaggrin and involucrin showed high upregulation on a protein level when cultured in systems with hydrocortisone containing supplements such as the HKGS confirming the differentiation of epithelial cell layers.

The development of a scaffold-free in vitro model of the oral mucosa has recently been described, using the TR146 carcinoma cell line with changing culture conditions as the model matured.^174^ Initially the culture was supplemented with HKGS which aids in the formation of a barrier membrane as described in previous models discussed within this review.^148,166^ Following 48 h incubation under these conditions’ cells were exposed to the air for 10 min to stimulate differentiation. A further 14 days incubation in air-lift conditions was then undertaken using media supplemented with calcium chloride to continue driving differentiation. Histological characterisation showed normal tissue attributions such as a cubic morphology of basal cells whilst the stratum intermedium displayed both cubic and polygonal cells with centrally located nuclei. The stratum corneum presented flattened cells with squamous morphology. The entire model had a thickness of about 150 µm and 8–12 single cell layers. Immunohistochemical staining showed that the model produced by this group represented a non-cornified stratified epithelium as CK-13 and CK-14 were abundantly expressed in the stratum intermedium and basal respectively.

**Figure 3.**
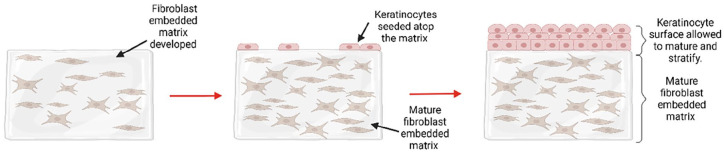
The general steps described to produce most of the in vitro mucosal models described throughout the literature, involving the infiltration of a 3D matrix with fibroblasts followed by subsequent maturation and topping with keratinocytes which are allowed to mature, stratify and cornify to produce a barrier epithelium.

Given the information presented here it appears no matter the biomaterial support structures used for the model, there are several common themes throughout the literature in regard to producing clinically relevant in vitro oral mucosal models (Figure 3). Whilst early models focussed on the harvesting of primary cells from tissue biopsies, in recent years there seems to have been a shift in practice to use immortalised cell lines. The benefit of this is that it enhances model reproducibility, which is sacrificed when using primary cultures due to donor-donor variability. The culture conditions throughout the model development and maturation have a significant effect on the production of a barrier type membrane similar to that found in the in vivo oral mucosa. Evidence presented here suggests that the most important considerations to make here are the media supplementation during keratinocyte culture development, and exposure of the model at the air-liquid interface. Both factors appear to play a role in driving the differentiation of the keratinocytes and formation of the barrier layer important for investigations such as drug permeability studies.

### Disease states

Whilst mucosal models of healthy oral mucosa are ideal for testing permeability and diffusion rate of drugs intended for delivery systemically via the blood stream, in cases of local delivery to injury/infection sites of the oral mucosa itself these models would not be suitable representatives of the target anatomy. Therefore, it is necessary to consider the development of disease state models to satisfy these testing requirements.

#### Periodontal disease

Many examples of ‘periodontal pocket’ models can be found within the literature however most of these are simply a biofilm model in close proximity to a monolayer structure of gingival epithelial cells.^175,176^ Whilst this model is appropriate for some studies such as assessing the effects of biofilm formation, removal or treatment on the production of inflammatory stress markers by the epithelial cells it does not satisfy the requirements of using the model to assess infected mucosal permeability in drug delivery investigations.

One group have developed a 3-D model resembling the periodontal pocket anatomy allowing the simultaneous interaction between gingival tissue, immune cells, and oral biofilms.^177^ Here a perfusion bioreactor set-up was used to co-culture immortalised human gingival epithelial keratinocytes (HGEK-16) and immortalised human gingival fibroblasts (GFB-16). The GFB cells were expanded to infiltrate a collagen sponge creating a matrix representative of the stratum intermedium. Following several days culture, the HGHK cells were seeded atop the sponge until a continuous monolayer covered the 3D scaffold structure. The human myelomonocytic cell line, Mono-Mac-6, were injected to the system to represent immune cells in the oral tissues. Finally, discs inoculated with the biofilm were introduced to the perfusion chambers facing the epithelial surface of the 3D tissue model. The final model showed histological similarities to the periodontal pocket in vivo. The model system further displayed in vivo relevance via the upregulation of inflammatory cytokines IL-1β, IL-6, IL-2 and TNF-α in the model which was in contact with the biofilm compared to a control model. Increases in levels of these cytokines within the gingival crevicular fluid of patients presenting with periodontitis have been previously described.^178^ Whilst this model is closer to what is required to study the permeability of mucosal tissues in a state of periodontal disease in drug delivery studies the integrity of the tissue construct has not been fully characterised. In order to ensure the relevance of this model for use in drug permeability studies some membrane integrity studies such as TEER should be conducted and compared to native mucosal tissue presenting with symptoms of periodontal disease.

#### Precancer models

Dysplastic oral lesions have been shown to be precancerous in nature, the potential for these lesions to progress to develop tumours is highly dependent on exposure to several risk factors such as smoking, alcohol and tobaccos. Animal models of this nature are difficult to establish and yield unreliable results and cell lines which have been used for pharmaceutical research are likely to yield different results than more sophisticated multilayered tissue structures. Gaballah et al.^179^ presented a range of different conditions to produce in vitro epithelial tissues of varying levels of dysplasia resembling that of clinical lesions. Cells were isolated from clinical lesions displaying mild to severe dysplasia and combined with J2-3T3 fibroblasts incorporated within a collagen matrix. Keratinocyte strains which have immortal or extended lifespan showed the most reproducible models of mildly (DOK cells), moderately (D20 and POE9n cells) and severely (D6 and LDOK) dysplastic oral tissue whilst those derived using mortal dysplastic keratinocytes showed unpredictable phenotypes which did not necessarily match in vivo clinical lesions. These models have been applied to the study of new treatments using viral lysis to treat oral precancerous mucosa as an alternative to surgical tissue removal.^180^ Despite differences in the proliferative capabilities in the model epithelial tissue made from mortal cell lines, all other studies indicated that these models of oral dysplasia demonstrate close similarity to clinical lesions.

#### Ulcerative state

The need for the development of a 3-D in vitro tissue model of ulcerative oral mucosa was first described in 2011 by Lambros et al.^181^ as the oral mucosa is often left in a state of mucositis following chemotherapy or radiation treatment. Therefore, there is a need for a reliable method of in vitro testing of new therapies for mucositis treatment. During initial studies this group used a commercial 3-D oral tissue model (EpiOral^®^, MatTek, Ashland, MA) with exposure to gamma irradiation at a range of doses used to induce the ulcerative state. Histological analysis showed significant morphological changes following irradiation at 12 Grey (Gy). This dose caused loss of tissue coherence with areas of tissue displaying cells with swollen morphology which had lost their polarity. Using a TUNEL assay apoptosis was found to be abundant in samples irradiated with 12 Gy whilst tissues exposed to 2 Gy irradiation remained comparable to controls both histologically and regarding number of apoptotic cells. Additionally, the gamma irradiation caused alteration in several inflammatory cytokines and genes related to the NF-κβ pathway. The group have since successfully used this model in studies investigating the efficacy of treatments for oral mucositis.^182,183^

Colley et al. describe a similar method of inducing an ulcerative state in their tissue engineered oral mucosal model.^184^ The model was established using a matrix seeded with normal oral fibroblasts and human dermal microvascular endothelial cells with NOKadded after 72 h incubation of the initial co-culture. Following a further 24 h incubation the models were raised to the air-liquid interface. Experimental models were exposed to a single dose of 20 Gy irradiation. Changes in cytokine expression at day 7 and 14 were similar to that observed by Lambros et al.,^181^ however at day 21 there was a general decrease in cytokine expression across all models (sham and irradiated). This study continued follow-up experiments post irradiation for a longer period than those previously described. Decreases in cell viability, epithelial damage, keratinocyte apoptosis and decreased proliferation were maintained in the irradiated model up to day 21 post irradiation.

Given the observation that treatment with chemotherapy drugs often leads to the development of oral mucositis, Sobue et al.^185^ investigated the treatment of in vitro oral mucosal models with a chemotherapeutic to induce characteristics of oral mucositis. The model comprised a collagen I matrix embedded with fibroblasts, overlayed with human oral keratinocytes. The construct was matured over 2 weeks at the air-liquid interface to facilitate epithelial differentiation and stratification. The 3-D constructs were exposed to doses of 1 or 10 µM 5-fluorouracil (5FU) for 16 h. The treatment of models with 10 µM 5FU caused alterations to the model which represented that of in vivo ulcerated tissue. Characterisation of the model treated with the chemotherapeutic drug showed inhibition of DNA synthesis, cell apoptosis and stimulation of key proinflammatory cytokines. Histologic investigation showed the widening of paracellular spaces accompanied by significant increases in LDH release when treated with the higher 5FU dose. A lack of BrdU-positive cells in the treated models were indicative of a lack of DNA synthesis following chemotherapeutic treatment. Additionally, treatment with the drug caused a dose dependent increase in percentage of active caspase-3 positive cells representative of the apoptotic cells in the model.

Most recently El-Howati et al.^168^ have described the successful generation of engineered inflammatory oral mucosa which resemble the in vivo oral lichen planus ulcerative condition. The oral mucosal model designed here was based off that already described by Jennings et al.^167^ with modifications to include CD4+ Th- and CD8+ T-cells, which are observed in abundance in ex vivo oral lichen planus biopsies. T-cell culture was optimised to ensure activation and polarisation towards these specific phenotypes. These cells were loaded into a rat tail collagen hydrogel and following 7 days maturation of the NOF/FNB6 co-culture the normal oral mucosal model was transferred onto the top of the T-cell loaded hydrogel. A collagen solution was used to adhere the two hydrogels and after a further 3 days culture of the T-cell model the epithelium was stimulated with TNF-α and IFN-γ to induce cytokine production. This method produced three distinct cell layers which could be observed using H&E staining; the epithelium, a densely populated fibroblast matrix and a band of inflammatory T-cells contained sub-epithelially. Following stimulation with TNF-α/IFN-γ it was obvious both histologically and via analysis of T-cell specific cytokines that the T-cell layer greatly increased epithelial destruction towards that seen in vivo during oral lichen planus. Histological staining showed recruitment of the T-cells, within the T-cell model, towards the epithelium with high levels of T-cell infiltration in the fibroblast layer, coupled with apoptosis of basal/suprabasal layers and liquefactive necrosis of the basement membrane. These features were comparable to those seen in clinical biopsies of oral lichen planus. Additionally, this study showed the potential for application of the model to pharmaceutical validation using both a drug solution and a novel drug delivery patch.

#### Fungal and bacterial

**Figure 4.**
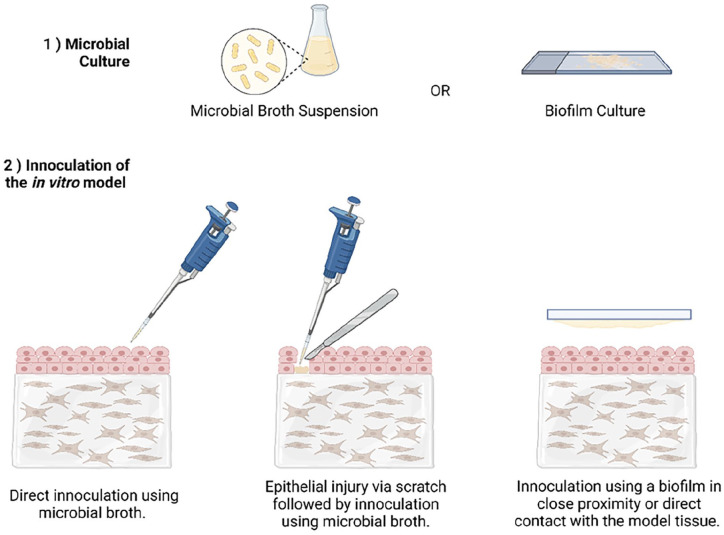
Common methods of producing infection models of the oral mucosa for in vitro studies. Three methods have been frequently described for the infection of in vitro model tissue with fungal and bacterial species associated with common oral infections.

Long term co-culture of bacteria or fungi with an in vitro oral mucosa model is challenging as the two require different culture media. Consequently, common practise appears to be to culture the in vitro mucosal model separately from the bacteria or fungi with subsequent infection of the mucosal model. Several different modification methods have been described for the introduction of fungal or bacterial species to in vitro mucosal models. Most studies describe the infection of an in vitro model one of three ways: via introduction of a small amount of bacterial/fungal suspension to the surface of the model, infection using a biofilm in close proximity of the model, or via creation of a scratch in the epithelial portion of the model with subsequent fungal/bacterial infection (Figure 4). Whilst over 85 species of fungi can be isolated from the oral cavity the most frequently observed clinically and therefore most frequently studied in vitro is the *Candida* genus. Regarding bacterial infection the most common disease of the oral cavity is periodontitis which is a disease encompassing infection with a wide flora of bacterial species, the most abundant of which is the gram-negative, anaerobic strain *Porphyromonas gingivalis (P. gingivalis)*.

##### Fungal infection models

An in-house tissue engineered in vitro model comprised of a fibroblast embedded collagen matrix overlayed with oral epithelial cells, both of which were primary isolates from a tissue biopsy has been used in conjunction with the original clinical isolate of *Candida albicans (C. albicans)*.^186^ These were initially cultured separately, when the models reached full stratification, they were inoculated with the *C. albicans* at a concentration of 1 × 10^5^/cm^2^. Control and infected models were maintained for up to 24 h with analyses conducted at 2, 4, 8 and 24 h. Contact with *C. albicans* significantly increased expression and production of laminin-5 and collagen IV. Expression of MMPs which are involved in the degradation and remodelling of ECM was upregulated at various timepoints. Additionally, TIMP-2 secretion was significantly decreased in the presence of *C. albicans* suggesting that there is a compromise in the basement membrane tissue integrity following infection.

Another group using an in vitro mucosal system similar to that used by Mostefaoui et al. and a similar infection method of *C. albicans*, both live and heat inactivated, investigated the cytokine expression during infection to elucidate the contribution of oral epithelial cells in the local defence against *C. albicans*.^187^ Results showed high levels of IL-1β mRNA expression during early infection, followed by a significant decrease in expression at 8- and 24-h post-infection whilst at 48 h the expression was again increased. These results correlated with protein production as quantified using a western blot. This study is suggestive of a role for IL-1β in local and systemic defences against *C. albicans.* It is hypothesised that oral epithelial cells attempt to control the growth of the *C. albicans* during initial stages of infection but soon become overwhelmed by the fungus.

One group have provided a comparison between the use of a commercial in vitro mucosal model and in-house developed models in the subsequent production of models of *C. albicans* infection.^188^ Histopathologic changes following fungal infection in all models proved like that observed in vivo. Observed tissue damage included degradation of the epithelial layers and widening of the transcellular spaces in the basal layer. All models infected with *C. albicans* showed significant increases in lactate dehydrogenase (LDH) secretion, indicative of decreased cell viability in all infected samples.

An investigation into the infection of a mucosal model, comprising NOK seeded atop a matrix of normal oral fibroblasts embedded in collagen, using 12 different strains of *C. albicans* has been provided by one group.^189^ The authors aimed to characterise the effect of *C. albicans* infection on E-cadherin functionality in oral epithelial cells. Whilst the gene expression of E-cadherin was not altered by the fungus, it did appear to be proteolytically degraded in localised areas of fungal invasion. It was confirmed that tissue invasion is necessary to stimulate the degradation via protease action as an invasion deficient strain of *C. albicans* failed to cause degradation of E-cadherin.

In other studies researchers have utilised commercial in vitro models of the oral mucosa as a basis for forming infection state models. Green et al. infected the SkinEthic model using a suspension of *C. albicans* (2 × 10^6^ cells).^190^ Co-culture of the model with the fungal species caused destruction of the epithelial layer and biofilm formation on the tissue surface, as observed via light microscopy. However, this study provided more insight into the effect of interaction with the mucosa on the *C. albicans.* Results from RT-PCR showed that expression of ALS genes by fungi interacting with the tissue engineered mucosa did not significantly differ from the expression in the original cultures used to inoculate the models. Silva et al. demonstrated the same mechanisms of tissue invasion by fungal species.^191^ However, in this model it was demonstrated that the invasive nature of *C. albicans* is not matched by all fungal species found in the oral cavity. For example, *Candida glabrata (C. glabrata)* did not demonstrate any tissue invasion of the RHOE model from Skinethic Lab^®^ (Nice, France). Whilst dual infection with *C. glabrata* and *C. albicans* demonstrated more extensive tissue damage than *C. albicans* alone. Therefore, it is concluded that *C. albicans* can not only cause extensive tissue damage on its own, but it can also enhance the invasiveness of other species. Whiley et al. demonstrate similar results showing that fungal penetration into the submucosa is species dependent.^192^ Yadev et al.^193^ compared the histological changes in two commercially available oral mucosal models following *C. albicans* infection to native infected tissue. The model based on NOK (EpiOral™, MaTek, Ashland, MA) showed histological changes closer to that of the infected in vivo tissue than the models based off a carcinoma cell line (RHOE, Skinethic Lab, Nice France). Both models displayed tissue invasion by the fungal species, however, the cytokine response was attenuated in the model containing NOK.

##### Bacterial infection models

Bacterial models have been previously described using simple epithelial monolayer cultures but recently it has become appreciated that to improve clinical relevance of studies 3-D organotypic models are more desirable. *P. gingivalis* is frequently investigated alongside the oral mucosal models as this anaerobic gram-negative strain is heavily implicated in the development of periodontal diseases. One study has investigated differences in cytokine expression between a normal oral keratinocyte monolayer and a normal oral keratinocyte 3-D mucosal model following infection with *P. gingivalis*.^194^ A cytokine blot revealed some differences in cytokine release between the two following infection with *P. gingivalis*, similar expression of IP-10, TIMP-2 and TNF-α was observed between the monolayer and 3-D culture. However, there was marked differences in a number of cytokines between the two experimental models. For example, IL-8 appeared to be downregulated following infection in the monolayer model whilst the mucosal model showed an upregulation of this cytokine. The opposite is true for IL-1α expression, this appeared upregulated in the monolayer culture and downregulated in the mucosal model. This study highlights the significance of designing 3-D in vitro models for testing to replicate the in vivo environment as closely as possible.

A model of the oral mucosa which incorporated a fully functional biofilm was first reported by Ryck et al.^195^ in 2014. The group analysed the crosstalk between the biofilm and the oral mucosal model using a modular model set-up allowing separate analysis of the biofilm and the mucosal model. Several interactions between the mucosal model and biofilm were observed which suggest the co-culture causes a bi-directional negative effect on the physiological properties of both components of the model. Presence of the mucosal model negatively impacted the number of bacterial cells residing in the biofilm, additionally, the size, complexity, and diversity of the biofilm changed when co-cultured with the mucosal model. On the other hand, a wound healing study showed the effect that the presence of the biofilm had a negative effect on wound recovery at 48 h in both complex models using either TR146 tumour derived cell line or a human keratinocyte derived from a non-tumorigenic source (HaCaT). This model is advantageous as the filter separation of the microbial biofilm from the complex mucosal model facilitates the independent analysis of the biofilm from the mucosa. This model could be applied to in vitro studies of drug efficacy in infected mucosa, however despite the advantages of the physical separation of the biofilm from the engineered mucosal tissue this may limit the application of this model for some studies.

Given that the failure of dental implants is often due to bacterial colonisation and biofilm formation on material surfaces it follows that the development of in vitro models representative of such interactions have been investigated. This has been described using an organotypic oral mucosa engineered using oral keratinocytes seeded atop a collagen embedded fibroblast matrix, implant material (titanium) and oral biofilm comprising either *Steptococcus oralis (S. oralis)* or *Aggregatibacter actinomycetemcomitans (A. actinomycetemcomitans)*.^196^ The mucosal model created an intact tissue-implant interface representative of what is seen following in vivo implant placement. For infection of the mucosal model, it was exposed to the biofilm for 24 h with effects of the biofilm exposure analysed histologically showing loosening of the mucosal tissue at the implant interface following infection with *S. oralis.* Therefore, it is concluded that molecular interactions during microbial infection at the implant-tissue interface are species specific.

In following years, the same model used by Ingendoh-Tsakmakidis et al.^196^ was modified to incorporate a multispecies biofilm which is more representative of the in vivo microbiome.^197^ The multispecies biofilm incorporated *S. oralis, Actinomyces naeslundii (A. naeslundii), Veillonella dispar (V. dispar)* and *P. gingivalis.* The study demonstrated at 24 h post infection the host-microbe homoeostasis was maintained however this became disrupted following a further 24 h incubation. The initial maintenance was due to a protective pro-inflammatory response by the in vitro mucosa model as evidenced by elevated secretion of inflammatory cytokines. However, sustained enhanced expression of these pro-inflammatory cytokines leads to tissue damage, which was observed histologically in the study showing tissue damage and mucosal detachment from the implant surface at 48 h post infection. These results mimic those previously described during clinical studies of cytokine secretion in periodontitis patients.

##### Dual infection models

Diaz et al. produced an infection model incorporating both fungal and bacterial strains, *C. albicans* and *S. oralis, S. gordonii* or *S. sanguinis* to replicate the in vivo scenario of interaction between the two organisms which promotes the mucosal colonisation and infection.^198^ Mono-species and mixed species biofilms on top of the engineered mucosal model were produced in a flow chamber. This set-up allowed the production of the biofilms under salivary flow which better mimics the in vivo conditions of biofilm formation than static cultures such as those previously described. Presence of *Streptococci* in biofilms increased the invasion of the oral mucosal model by *C. albicans.* Significant increases in *C. albicans* invasion were recorded at 24 h post infection using immunohistochemistry and confocal microscopy. Additionally, it appeared that the presence of *Streptococci* increased the biomass of *C. albicans* in the mixed species biofilms.

Similarly, a model produced using a biofilm of *C. albicans* and *S. aureus* on a tissue engineered oral mucosal model comprising a fibroblast embedded collagen matrix overlaid with a suspension of NOK which were developed until monolayer formation.^199^ The model was then matured at the air-liquid interface for 14 days. The oral mucosal models were then infected using mono-species or dual species microbial suspensions. Histological evaluation showed that the *C. albicans* alone was capable of infiltrating and infecting the epithelial layer of mucosal tissue whilst the *S. aureus* was not. By comparison, infection by the dual species biofilm caused more extensive damage than the *C. albicans* alone. This damage, by the dual species infection, extended deeper into the subepithelial space. These results were also reflected in an LDH assay where the lowest levels of LDH increase compared to uninfected controls were observed by the *S. aureus* infection, whilst the dual infection presented the highest level of LDH. The results confirm the hypotheses that some bacterial species are incapable of penetrating barrier surfaces and require another species to breach the surface to facilitate their invasion of the tissue.

Bertolini *et al.* have described the polymicrobial infection of a mucosal injury model similar to that produced by Sobue et al. in 2018.^185,200^ There was an enhanced fungal invasion of injured tissues dual-infected with both *C. albicans* and *E. faecalis.* Pretreatment of the mucosal model with 5-FU to induce mucosal injury caused increased susceptibility to invasion by *C. albicans* and both pathogens in the dual-infection model. These results resembled those obtained via fluorescent labelling of oral bacterial and fungal infection in the absence or presence of 5-FU treatment during an in vivo study using tissue sections of murine tongue.

Most recently, Gould et al. have described an investigation into the in vitro release of pro-inflammatory cytokines by an in vitro oral mucosal model.^201^ *S. aureus* and *C. albicans* elevated the production of pro-inflammatory cytokines by in vitro models upon infection but did not exhibit any enhanced effect when co-cultured. These results did not align with reports using an in vivo murine periodontitis model which descried elevation of pro-inflammatory cytokines, IL-6, GCSF, KC MCP-1 and MIP1α, during a co-infection medal compared to individual infection of *C. albicans* or *S. aureus.* It is possible that this is due to the lack of immunomodulatory cells present in the in vitro model which play significant role in cytokine secretion in vivo. Whilst this model showed histological similarities to in vivo mucosal tissue infected with *C. albicans* and *S. aureus* and is a useful advancement in the development of in vitro mucosal models; to enhance the clinical relevance and enable the study of mechanisms of inflammatory responses, it may be important to introduce immunomodulatory cells such as neutrophils or macrophages.

#### Concluding remarks on *in vitro* mucosal disease models

Contact time of the infection species with the mucosal model was a maximum of 48 h across all the studies, this is likely due to complications in finding a suitable growth medium for successful long-term co-culture. Without any suggestion of a resolution for this limitation in the development of infection models, a significant restriction is put on the use of these infection models as drug delivery testing platforms. Whilst it is useful to have a complex in vitro tissue platform to use for testing the initial effects of drug interaction, many treatments are required for extended periods. Therefore, realistically to monitor the effects of drug interaction an in vitro platform which can meet these longer-term testing requirements would increase the clinical relevance of the test model.

Elsewhere research has shown interesting results on maintenance of organoids in hypoxic conditions,^202^ given that many of the organisms associated with the oral microbiota are anaerobic strains this is possibly an avenue of research which should be applied to the development of oral mucosal models. Advancement of oral mucosal modelling to incorporate such features could also enhance the possibility for disease modelling and translation into long-term drug discovery experiments.

Additionally, many of the models investigated as in vitro replicas of mucosal infection did not report quantitative results on the integrity of the barrier membrane following infection. If these models are to be used in the study of drug delivery across the oral mucosa this is a factor which should be investigated and compared to in vivo tissue in the same state of infection. The study by Villar et al.^189^ describes the degradation of E-cadherins following infection by *C. albicans*. These proteins are integral in contributing to the maintenance of the epithelial barrier functions via regulation of the claudins into tight junctions.^203^ Therefore, it is a reasonable assumption that the barrier function is reduced by *C. albicans* infection. However, this should be further investigated using previously discussed methods such as TEER measurement to develop a more robustly characterised mucosal infection model.

Moreover, the lack of presence of immune cells in currently developed in vitro models has been recognised. To improve the clinical translation of these models it may be advantageous to utilise a mucosal model which incorporates immunomodulatory cells, such as that described by Bao et al.^168^ and El-Howati et al.^177^ in conjunction with some of the infection methods described in the studies presented here.

## Ex vivo modelling techniques

The oral mucosa is distinguished by superior drug accessibility, quick absorption due to relatively high blood flow, a robust epithelium, bypass of first-pass metabolism, and less exposure of medicines to the GI environment^34,204^ which makes it an important route for drug delivery. However, the complex structure of oral mucosa possesses a critical barrier for mucosal drug delivery. Different studies have shown that the outermost layer is the main barrier to drug diffusion, while the underlying layers are relatively permeable.^205,206^ Therefore, according to the specificity of the route of administration and more detailed knowledge of the composition of the oral mucosa, a considerable advancement has been made during the last decades in therapeutic DDSs designed to sustain a novel approach for the treatment of a wide number of disorders.^207^

### Differences between in *vitro* and *ex vivo* modelling

Given the complexity of oral mucosal drug delivery, ex vivo oral mucosa models (derived from animal mucosae) or in vitro models (obtained utilising cell cultures) have been extensively studied in the recent era. However, a standardised system reproducing oral mucosa properties, allowing a rational synthesis of pharmaceutical formulations resistant to salivary flow, movement of the tongue, and chewing, is highly desirable and not yet available.^148^ Nevertheless, numerous ex vivo and in vitro models are currently used depending on the investigations being carried out,^208^ with each model offering certain advantages and disadvantages for evaluating the permeability of the drugs (Table 2).

**Table 2.** Comparison of in vitro and ex vivo models.

Model Feature	In vitro	Ex vivo
Source	Tissue specific primary cells or immortalised cell lines.	Tissue explants from human or animal
Spatial Structure	In vitro cultures can be prepared in 2D and 3D arrangements. 3D cultures resemble skin tissues.	3D structure like live skin tissues.
Biological responses to a treatment or stress	More reactive due to the simplified nature of the model.	Less reactive due to the robustness and complexity of the model.
Lifespan	Limited, with primary cell cultures and reconstructed models usable for a few days to a few weeks during the maturation period.	Used within a period of 10–14 days based on culture conditions.
Diseased models	Mimicked in the laboratory through chemical /biological stimuli, making them available as needed.	Real diseased tissue but dependent on sourcing and supply availability.
Genetic Engineering	Extensive. Targeted genetic engineering is possible in isolated cell populations (gene knockout, transgene, CRISPR).	

In vitro model systems often fail to consider the extremely complex microenvironment, a large part of which remains unexplored. In vivo model systems have been invaluable tools to validate and complement in vitro findings. Yet, they are more expensive, low-throughput and their translatability is still debatable due to species differences. These limitations have prompted to develop ex vivo systems, which sought to decrease the knowledge gap between in vitro and in vivo models. One important advantage of ex vivo systems over traditional in vitro systems or even organ-on-a-chip systems is that it preserves the surface topography and 3D architecture of the native tissues. A growing body of evidence clearly supports that nano- and microscale surface topography has a huge influence on both bacterial attachment and bacterial signalling in the ex vivo modelling of mucosal infectious disease such as biofilm formation.^209,210^ For instance, using a microfluidic device to control spatial structure and chemical communication, it was found that stable coexistence of interacting bacteria requires a defined microscale structure.^211^ Recent advances in material sciences also revealed that a reduction of bacterial adhesion can be achieved via the control of surface topography,^212^ further confirming the role of physiochemical regulation of biofilm formation. Lastly, the use of ex vivo tissues allow experiments to be performed in a more physiologically relevant environment that would otherwise be restricted from using in vivo models due to ethical issues. For these reasons, many ex vivo biofilm model systems are developed using tissues from both animals and human donors, including ex vivo dental and oral mucosal models. As a disadvantage, maintaining ex vivo models for a prolonged period is still a challenge. Depending on the size and geometry of the ex vivo tissues, an adequate supply of nutrients and oxygen throughout the tissue may also be an issue.

This narrative review’s objective is to discuss the state-of-the-art oral mucosal models – which are not commercially available – used to assess the DDSs, paying special attention to more ambitious modelling techniques for different disease states. In this regard, efforts were made to choose more appropriate models based on the effect that needed to be determined.

### Tissue harvest sources

Ex vivo samples derived from animal buccal tissues are often used as models for human buccal epithelium, as tissues of human origin are rare. Among animals, oral mucosa is mostly obtained from pigs, rats, hamsters, rabbits, dogs, and primates.^208^ All these models present specific limits mainly related to characteristics such as thickness and keratinisation, even if they are acceptable for studying the trans-buccal absorption of selected drugs. Obviously, it is important to consider these differences during analysis to properly compare these results for human applications (Table 3). It is evident that using different animals to obtain oral mucosa samples is the first reason for the lack of a standardisation in ex vivo modelling; nevertheless, other factors also hinder this aim. In fact, when mucosa derived from the same animal species is used, the following factors can also hinder standardisation: different cell culture conditions, a limited amount of tissue from the cheek, and the intrinsic instability of oral mucosa due to the stress that the animal undergoes before slaughter.^213^ Regardless, all these variants, both in the origin and in the preparation of the tissues for the models and in the experimental techniques, prevent the standardisation of ex vivo permeation studies.^214,215^

**Table 3.** Characteristics of different oral mucosa models.

Animals	Epithelium type	Advantages	Disadvantages
Rats	Keratinised		Different permeability^216^
Hamsters	Keratinised		Different permeability^216^
Rabbits	Non-keratinised or keratinised	The permeability resembles to human mucosa	The amount of mucosa is significantly reduced^217^
Dogs	Non-keratinised		The epithelium is thinner with respect to human one, thus the permeability is different^217^
Monkeys	Non-keratinised		The epithelium is thinner with respect to human one, thus the permeability is different^217^
Pigs	Non-keratinised or keratinised	The permeability values are like human mucosa^218^	

Consequently, it is very difficult to obtain adequate quality control regarding the evaluation of permeability and cell viability, the latter of which is essential for maintaining the barrier ability in mucosa models. Cell viability is generally determined by 3-(4,5-dimethylthiazol-2-yl)-2,5-diphenyltetrazolium bromide assay (MTT) at the beginning of the permeation tests (which take several hours) and not at the end of the tests.^215,219^ To solve this problem, in a recent paper,^220^ the authors utilised an MTT assay to evaluate the mucosa viability before and after permeability tests, thus comparing the cell viability of five different mucosal models extracted and preserved under different modalities and conditions. In particular, the media utilised were phosphate buffer solution (PBS), Kreb’s bicarbonate Ringer’s solution (KRP), KRP + 1% foetal bovine serum (FBS), and KRP + 1% FBS in a CO_2_ atmosphere. The mucosa ex vivo models were rats, rabbits, dogs, pigs and humans. The separation of the epithelium from the underlying connective tissue by heat treatment resulted in an epithelial thickness of approximately 500 μm without compromising the permeability and integrity characteristics of all different mucosae. Specifically, the authors placed oral mucosa (used in the permeability experiments) on a 6-well plate and cut the sample. A solution of MTT was added to each well, and after 4 h of incubation, the cells were lysed, and the formazan crystals were solubilised by DMSO. Thus, the absorbance at 540 nm was measured in each well, and the viability was determined relative to fresh mucosa, which was assumed to be 100% viable.^220^ The obtained results confirmed that mucosae maintained their maximum integrity in KRP at 4°C for 36 h without using any other protectant. Moreover, the authors reported that in the presence of selected cryoprotectants (20% glycerol and 20% trehalose), the mucosae, which were frozen at −80°C and thawed at 37°C, exhibited preserved integrity and biological viability for 21 days. Therefore, this study aimed to identify the experimental conditions to standardise the process of isolating, maintaining, and determining the viability of mucosa and thus improve the accuracy of permeability studies.^220^

### Various *ex vivo* modelling techniques

#### *Ex vivo* permeability studies

The permeability of a drug is defined by the coefficient ‘Log P’, with this parameter being the partition coefficient of the selected molecule between aqueous and lipophilic phases, which are usually water and octanol.^221,222^ Accordingly, Log P is an intrinsic property related to the chemical structure of a drug and its ability to perform hydrophilic or hydrophobic interactions between a nonionised form of the drug and its medium.^221,222^

Several permeability studies have been used to assess the absorption kinetics and permeation profile of compounds through buccal mucosa and the chemical penetration of enhancers. In the last years, ex vivo permeability studies have been more extensively employed compared to the in vivo permeability studies, since these techniques have lower cost, the samples are easy to analyse and the experimental conditions (e.g. temperature, pH and osmolarity) are maintained during the experiment.^6,223^ The main animal model used in the ex vivo permeability studies is the porcine buccal mucosa, due to the close similarity of this tissue with human buccal mucosa in terms of morphology, structure, composition and enzymatic activity.^224–226^ The porcine buccal tissue needs to be used for those permeability studies immediately after the slaughter of the animal, since the viability and integrity of the tissue is time-dependent.^227^ However, when it is not possible the dissected tissue needs to be stored at specific conditions.^208^ According to the literature, the viability and the lipid permeability barrier can be maintained using cryoprotectant agents, after the harvest of the tissue.^228^

Most of the buccal permeability studies are performed ex vivo, owing to their several advantages over in vivo studies. Indeed, ex vivo studies are less expensive, since only small pieces of dissected buccal mucosa are used and the experimental conditions (e.g. pH, osmolarity and temperature) are maintained during the experiment. The sample analysis is also easier, because the receptor solution is a buffer solution instead of a blood sample. Furthermore, these studies are less time-consuming and easily set up using different types of diffusion apparatus.^6,223^

Diffusion cells are widely used to determine the amount of drug that permeates the buccal mucosa and the rate of drug diffusion.^219^ The buccal mucosa from an appropriate animal is placed mounted in different types of diffusion cells: vertical diffusion cells (Franz diffusion cells), flow-through diffusion cells and side-by-side or horizontal diffusion cells (Ussing chambers and Sweetana-Grass chambers).^229^ All these systems are composed of a donor compartment, a receptor compartment, a sampling port and a heater with a temperature of 37°C. The physiological solution and the drug solution are introduced in the receptor and donor compartments, respectively.^96,230^

The accumulative amount (dQ) of compounds that permeated the buccal mucosa is calculated as per the following equation:

$$J_{ss}=\frac{dQ}{dt}\times \frac{1}{A}$$

Where *J*_ss_ is steady state flux, and *A* is the surface area of buccal mucosa available for permeation. The permeability coefficient (*P*_app_) can be calculated from Fick’s first law of diffusion ( $J_{ss}=P_{app}\times \Delta C$ ), considering that *C*_donor_ ⩾ *C*_receptor_ (receptor concentration).

Therefore, *P*_app_ is determined by the following equation:

$$P_{app}=\frac{J_{ss}}{C_{0,donor}}\times V_{receptor}$$

Where *C*_0,donor_ is the initial donor concentration, and *V*_receptor_ is the volume of the receptor compartment.

##### Franz diffusion cell

**Figure 5.**
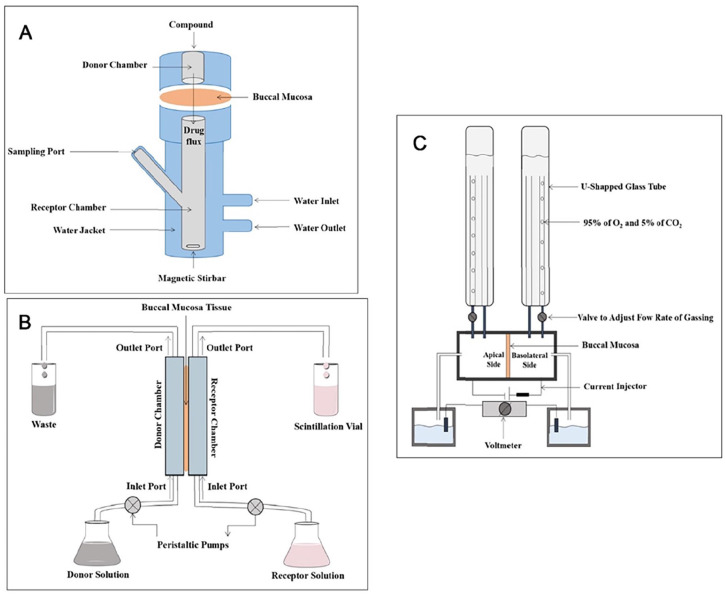
(a) Schematic representation of a Franz diffusion cell, (b) schematic representation of a flow-through diffusion cell, and (c) schematic representation of a circulation Ussing chamber.

The Franz diffusion cell (Figure 5(a)) is considered a static, one-chamber diffusion cell^208^ extensively used to evaluate the penetration/permeation of molecules through buccal mucosa, using tissues from pigs.^231–233^ The buccal tissue is assembled between both compartments, with the epithelium facing the donor compartment and the connective tissue facing the receptor compartment.^234–236^ The receptor compartment has a volume capacity of around 6–8 ml. This side of the cell has a stirring bar responsible for maintaining the temperature and mixing the receptor fluid. The temperature of the system remains at 37°C to mimic the temperature of in vivo environment. This temperature is achieved by placing the apparatus into a heated water bath.^237,238^ The samples from the receptor compartment are periodically withdrawn by the side port of the diffusion cell and the amount of drug that permeates the buccal tissue is determined. The volume is then replaced with a fresh buffer to maintain the experimental conditions.^230^

The Franz diffusion cells require a limited volume of receptor solution, which may be a problem when the tested drug has poor solubility in the receptor solvent. A co-solvent (e.g. ethanol, methanol and polyethylene glycol) can be added to the receptor fluid to improve the permeability and solubility of the drugs.^239^ In addition, the buccal mucosa placed in this apparatus is continuously exposed to the air. Therefore, the viability of the tissue could be compromised because of drying and the tissue death.^208^

##### Flow-through diffusion cell

Flow-through diffusion cell (Figure 5(b)) is commonly applied for drugs with poor solubility in the receptor fluid. The larger capacity of the donor compartment ensures a suitable loading of the drug solution, while the lower volume of the receptor compartment allows a rapid clearance of the penetrant at low pumping rates. After assembling the tissue, the diffusion cell is closed and the chamber is placed in a vertical position on a heated aluminium-holding block, maintaining the temperature at 37°C. One tube is attached to the inlet port of the receptor side to receive the receptor solution by a peristaltic pump. A glass chamber is included into the donor chamber and its inlet port is connected to the drug solution. Then, the glass donor chamber is filled with the drug solution. The donor and receptor solutions are perfused in the same direction.^240^ The effluent from the receptor chamber is continuously collected for scintillation vials and used to determine the amount of compound that crossed the buccal mucosa at each time-point.^223^

Lestari et al.^240^ validated this method by assessing the permeability of three compounds (caffeine as model hydrophilic drug, triamcinolone acetonide and oestradiol as model lipophilic drugs) through porcine buccal mucosa and comparing the *J*_ss_ values with the Ussing chamber. According to the results, the cumulative amount of all three compounds was similar between both diffusion apparatuses. The *J*_ss_ values of the three compounds were not significantly different (*J*_ss_ caffeine = 1.35 ± 0.30 μg/cm^2^/h; *J*_ss_ triamcinolone acetonide = 0.053 ± 0.037 μg/cm^2^/h; *J*_ss_ oestradiol = 0.32 ± 0.14 μg/cm^2^/h) compared to the Ussing chamber (*J*_ss_ caffeine = 1.33 ± 0.26 μg/cm^2^/h; J_ss_ triamcinolone acetonide = 0.065 ± 0.020 μg/cm^2^/h; *J*_ss_ oestradiol = 0.28 ± 0.08 μg/cm^2^/h). The similar permeability parameters in both models suggest that the flow-through diffusion cell can be used for assessing the drug permeability across buccal mucosa.^240^

Flow-through diffusion cell is very similar to Franz diffusion cells, but in this method the buccal mucosa surface is not exposed to the air, avoiding the drying of the tissue and the potential tissue death.^223^ The receptor solution flows underneath the tissue, providing better experimental conditions and closer resemblance with in vivo blood circulation.^241^ The automatic stirring of the system reduces the time of the experiment and the costs and ensures no accumulation of compounds in the receptor compartment. The buccal mucosa is placed in the vertical orientation to minimise the potential for trapped air bubbles, since these bubbles are often encountered in horizontal flow through system, such as Franz diffusion cells.^223^

##### Ussing chamber

As described by Ussing et al.,^242^ the Ussing chamber is used to study the active transport of sodium in the skin, using frog as animal model. Currently, this methodology has been used to analyse the transport of molecules across buccal mucosa and evaluate the effects of chemical penetration enhancers on buccal permeability.^243–245^ This technique is composed of two-half chambers, a perfusion system, an amplifier and a data acquisition system. The two half-chambers when clamped together are separated by a piece of biological tissue.^223^ A set of two electrodes are placed on both sides of the tissue to record the potential differences across them, while another set of two electrodes allows the injection of a current to reverse the potential differences. The intensity of the current to be injected is determined by an electric clamp apparatus.^246,247^

The Ussing chamber can be classified as circulating chamber or continuously perfused chamber. The circulating chamber (Figure 5(c)) is composed by a U-shaped tubing system filled with carbogen gas (95% of O_2_ and 5% of CO_2_) and N_2_, providing the stirring needed to ensure a complete convection and maintain the viability of the buccal mucosa.^227^ Moreover, the U-shaped tubing system assures the same hydrostatic pressure on both sides of the chamber, avoiding tissue damage caused by bending of the tissue.^208^ This procedure is considered an advantage over the Franz diffusion cells and flow-through diffusion cells. On the other hand, the continuously perfused chamber has two half-chambers responsible for minimising the hydrostatic pressure and preventing tissue damage. A reservoir attached to the chamber by polyethylene tubes is used to deliver the solutions present on both sides of the tissue to the Ussing system. The valves may be used to maintain the flow rate. The temperature of the system is ensured using a water jacket heating water.^248^

In the Ussing chamber, a small amount of drug is used. The samples collected are analytically cleaned. Furthermore, the bidirectional drug transport across dissected tissue should be evaluated, because the drug can be added at both sides of the chamber. In this method is possible to determine the TEER, to assess the viability of the tissue.^248^ However, Ussing chamber induces a relatively low throughput and the amount of tissue available per animal is limited, which can compromise the drug permeability.^247,249^

##### Sweetana-grass diffusion chamber

Sweetana-Grass diffusion chamber is a device derived from the Ussing chamber. Grass and Sweetana^250^ validated this method by testing the transport of drugs (mannitol, L-glucose, D-glucose, methoxy inulin, naproxen and progesterone) across rabbit intestinal mucosa and comparing the permeability results to another diffusion cell, the Ussing chamber. Briefly, the tissue was assembled at the same manner that in the Ussing chamber but preheating the cells at 37°C. Then, the cells were placed in an aluminium block heater and the reservoirs were filled with warmed oxygenated buffer solution that circulates by a carbogen gas (O_2_/CO_2_) through the reservoirs. At different time-points, the samples were collected from the receptor chamber and immediately replaced with the same receptor medium. The permeability parameters showed no statistical differences in both methods. Furthermore, these authors demonstrated several advantages of the Sweetana-Grass diffusion chamber over the Ussing chamber. The diffusion cells have only one material type (acrylic) responsible for reducing the probability of leakage. On the other hand, the Ussing chamber has two or three different materials (glass from the reservoir, acrylic from the cell and the connecting tubing). The temperature of the system is easier to control, since the cells and the surface area of the device are heated at the same time, unlike the Ussing chamber that only the fluid of the reservoir is heated. The cleaning is faster and more efficient than the Ussing chamber. The fluid low is parallel to the surfaces of the tissue, reproducing exactly the in vivo conditions. The volume of the reservoir is lower than the reservoir of the Ussing chamber, allowing the quantification of drugs with low permeability. For these reasons, Sweetana-Grass diffusion chamber can be used as alternative to Ussing chamber in the assessment of drug that permeate mucosal tissue. However, this method is not so practical than the Ussing chamber since it requires the separation of the reservoirs from the heating/circulating bath. Moreover, this apparatus cannot be used for electrophysiologic measurements as Ussing chamber.^250^ Sutton et al. modified the method developed by Sweetana and Grass, mounting Ag/AgCl reversible electrodes in agar-filled pipette tips that were compression-fitted into the diffusion cell cap. This modification enables the evaluation of the integrity of the tissue by measuring the TEER and studying the effects of the permeability of the tissue in the absorption of penetration enhancers.^251^ This method has been currently used to assess the bidirectional transport of drugs through porcine buccal and intestinal mucosa.^252^

#### *Ex vivo* modelling mimicking diseased state

##### *Ex vivo* modelling for oral mucosal infection

The human fungal species *Candida albicans* is a commensal fungus commonly colonising human mucosal surface.^253^ The increasing emergence of strains of *C. albicans* resistant to commonly used antifungal agents has made the clinical management of candidiasis increasingly difficult and the need for improved drug therapies crucial.^254,255^ Therefore, identifying the mechanisms and cofactors behind the enhanced susceptibility to oral candidiasis in vulnerable populations would serve as a breakthrough in our understanding of the pathogenesis of oral candidiasis.

In this instance, an ex vivo murine model of oral infection was developed to investigate the protective effect of histatin-5 (Hst-5) against *C. albicans*.^256^ For these experiments, tongues were excised from sacrificed 8-week-old female CD-1 mice. Tongues were placed in the wells of 24-well tissue culture plates containing 1 × 10^7^ cells/ml of C. albicans in 1 ml PBS. In order to determine the dose-dependent effect of Hst-5 on *C. albicans*, tongues were infected in the presence of increasing concentrations of Hst-5 (50–500 mg/ml) as well as to assess the susceptibility of *C. albicans* to Hst-5 (150 mg/ml) at various cell densities, CFU counts from tongues infected with *C. albicans* were determined and assessed as percent killing by Hst-5. In addition to Hst-5, experiments were also performed to determine the protective effect of host saliva against *C. albicans* in which tongues were treated with purified saliva without Hst-5 and incubated at 35°C for 30 min in the presence of Hst-5 or saliva with gentle rotation. The tongues were further incubated with RPMI 1640 to allow the germination and hyphal production by adhering yeast cells.

**Figure 6.**
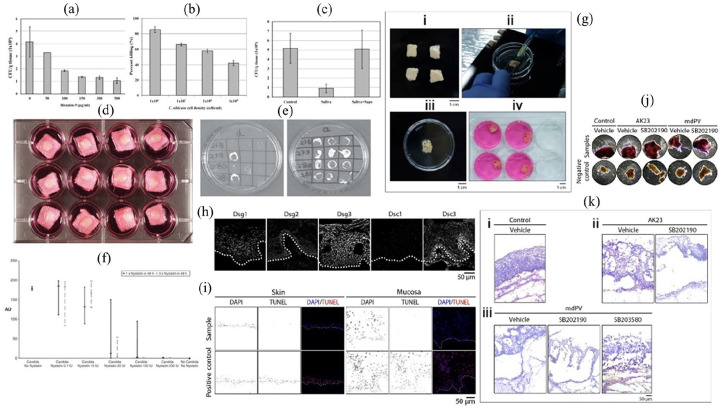
Protective effect of Hst-5 on *Candida albicans* viability (CFU/g tissue) using an ex vivo model of oral infection. (a) Significant decrease in *C. albicans* viable counts recovered from tongues infected in the presence of increasing Hst-5 concentrations demonstrating a dose dependent inhibitory effect for Hst-5 on *C. albicans*. (b) In contrast, the anticandidal effect of Hst-5 was inversely proportional to C. albicans cell density, where, based on the percentage of Hst-5 killing, the susceptibility of *C. albicans* to Hst-5 decreased with increasing cell density. Error bars indicate the SEs of the means. No significant difference is seen between 0 and 50 mg/ml Hst-5 concentration (*p*> 0.05). For all other values, *p* < 0.05. (c) A significant decrease in C. albicans CFU counts recovered from tongues infected in saliva (Saliva) compared with control tongues (Control) with PBS. However, no significant effect on CFU counts was seen when saliva was pretreated with purified proteases (Saliva + Saps). (d) Ex-vivo pig mucosa organ culture model (PMOCM). The photograph demonstrates 12 porcine mucosa organ culture models. (e) Photographs of imprinted agar plates. Imprints incubated for another 24 h which were made after termination of the infection culture by inverting the punch biopsies and pressing the treated skin onto the surface of agar plates (a: treatment with nystatin 20 IU, b: treatment with nystatin 0.1 IU). (f) Evaluation of fungal growth after 48 h in pig mucosa organ culture model. The graph demonstrates the medians of arbitrary units, after 48 h incubation. Error bars represent 95% confidence interval for the median. (g) Ex vivo human mucosa model (i) large specimen tissue, (ii) 30G syringe was allowed to pass through the mucosa sample along its long axis, (iii) Injection areas are marked and (iv) samples were allowed to float on DMEM with the mucosa facing upwards. (h) Immunostaining of desmosomal proteins to characterise mucosal explants (*n* = 3). Desmoglein (Dsg)3 shows a higher intensity staining across all layers, whereas Dsg1 shows a lower intensity staining that was missing in basal and suprabasal keratinocytes. In contrast, Dsg2 and desmocollin (Dsc)1 were almost absent, whereas Dsc3 was detectable by immunostaining in the basal and suprabasal layer only (*n* = 3). (i) Representative immunostaining of terminal deoxynucleotidyl transferase dUTP nick-end labelling (TUNEL) assay showing skin and mucosa samples treated with and without DNase I as positive control. Apoptosis was not detectable in samples indicated by negative TUNEL staining (*n* = 3). Dotted lines represent basement membrane. (j) A positive test for viability was observed as viable tissues induced a colour change from yellow to blue detecting mitochondrial activity; boiled negative control samples did not yield any colour change. mdPV, mucosal-dominant pemphigus vulgaris. (k) AK23 antibodies or mucosal-dominant PV (mdPV)-IgG were applied for 24 h in the absence or presence of p38 mitogen-activated protein kinase inhibitors SB202190 or SB203580, preincubated for 1 h. (i–iii) Representative haematoxylin and eosin staining of cryosections from samples.

**Figure 7.**
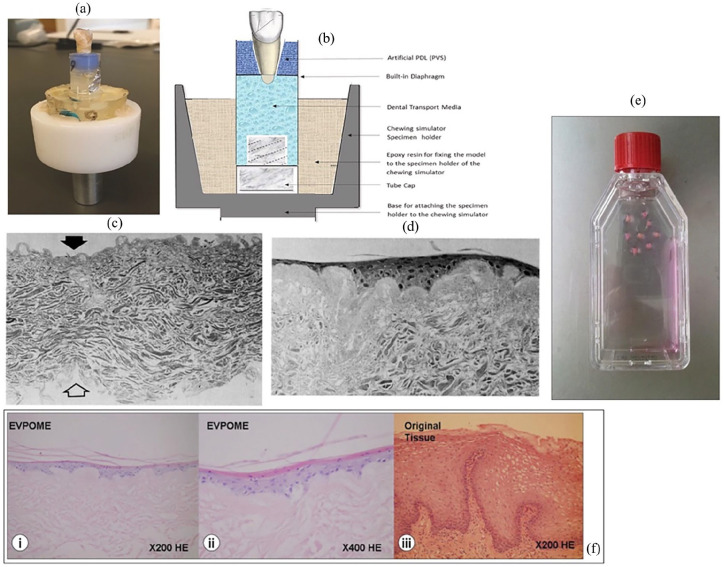
(a) Experimental model, (b) diagrammatic illustration of the model and (c) rehydrated AlloDerm. Closed and open arrows show the epidermal and dermal surface, respectively (haematoxylin and eosin, original magnification 100×). (d) Continuous stratified epithelial layer at a cell concentration of 5.0 × 10^4^ cells/cm^2^ cultured for 1 week at an air-liquid interface. Cells in the basal layer are cuboidal or polygonal. Cells in the superficial layer are flattened (haematoxylin and eosin, original magnification 250×). (e) The tissue specimens, approximately 1 × 1 mm in size, placed in a T-25 flask. (f) Histological appearances of the “EVPOME”s (i) 200× H.E. (ii) 400× H.E. and (iii) the original tissue sample (200× H.E.).

Results depicted a dose-dependent killing potency for Hst-5 based on decreasing number of CFU counts proportional to Hst-5 concentration (Figure 6(a)). However, the susceptibility of *C. albicans* to Hst-5 was inversely proportional to its cell density, where, based on percent of killing, higher *C. albicans* cell densities demonstrated decreasing susceptibility to Hst-5 (Figure 7(b)). Moreover, results from the experiments with human saliva were comparable to those obtained from Hst-5 treatment, where a drastic reduction was observed in the viable numbers (CFUs) of C. albicans from tongues treated with saliva (Saliva) compared with control tongues (Control) (infected in PBS) (Figure 7(c)). The saliva samples pretreated with proteases to deactivate Hst-5 activity were also investigated to further confirm the Hst-5 as a saliva component responsible for anticandidal effect of saliva. The result depicted that pretreated saliva samples resulted in almost complete loss of anticandidal activity (Figure 6(c)).

The antifungal activity of Nystatin against oropharyngeal infection caused by *C. albicans* was also evaluated by using porcine oral mucosa in ex vivo modelling technique.^257^ In this study, the oral mucosa was obtained from an intact porcine carcas immediately after death. All pigs were of the same breed (crossbred Yorkshire/Deutsches Edelschwein). Approximately 1 h after slaughtering the pig snouts were cleaned and disinfected and 8 mm punch biopsies were taken and placed dermis down on sterile gauze in culture dishes and immersed in medium such that the dermis was solely in contact with the medium while the mucosa surface remained exposed to air (Figure 6(d)). The DMEM media consisted of hydrocortisone, 5% foetal calf serum, penicillin and streptomycin. The resultant ex-vivo pig mucosa organ culture model (PMOCM) was incubated in ambient air with 10% CO_2_ at 37°C for up to 5 days. The PMOCM were inoculated with *C. albicans* and following incubation nystatin was administered on top of the punch biopsies. After termination of the infection culture, imprints were made by inverting the PMOCM and pressing the treated skin onto the surface of agar plates.^258^ The imprinted agar plates were incubated, and plates were photographed for documentation of organism growth (Figure 6(e)). Following the experimental procedure the result showed a significant reduction of fungal growth in the test groups with 230, 100 and 20 IU nystatin in single and triple application, imprint after 48 h (Figure 6(f)).

Additionally, a new human oral mucosal model was developed to investigate the role of p38MAPK in preventing autoantibody-induced mucosal blistering in pemphigus.^259^ Pemphigus vulgaris (PV) represents an autoimmune disease characterised by suprabasal blister formation due to loss of keratinocyte cohesion in stratified epithelia such as skin and oral mucosa that are subjected to substantial shear forces.^260,261^

In association to this, it was noted that desmosomes experience tension when keratinocytes are exposed to mechanical strain^262^ which caused the activation of autoantibodies (PV-IgG) against cadherin-type adhesion molecules in desmosomes particularly desmoglein (Dsg)1 and Dsg3.^263^ The mechanism involved in PV progression is associated with autoantibody mediated direct cell dissociation via inhibition of Dsg interaction and altered p38 mitogen-activated protein kinase (p38MAPK) signalling which leads to disturbed desmosome turnover and uncoupling from the keratin filament cytoskeleton.^264,265^ Recently it was demonstrated that p38MAPK inhibition was effective in attenuating blister formation, as well as in reducing desmosome size and number in human epidermis after PV-IgG injection into cultured skin explants.^266^ However, the inhibition of this signalling pathway is still a point of confusion as a feasible treatment approach for PV patients in clinical settings. In this regard, a clinical study using the p38MAPK inhibitor KC706 in an open-label trial with 15 patients with PV was not successful.^267^ Thus, Egu et al. and his colleagues developed an ex vivo model to characterise blister formation and ultrastructural alterations of desmosome morphology in the inner lining of the labial mucosa to test the relevance of p38MAPK.

The ex vivo human mucosal model for PV was developed from the mucosa biopsies from the inner lining of the lips from body donors without any history of oral lesions and only those bodies arriving within 12 h after decease were considered for the study. A strip of mucosa, approximately 2 × 6 cm, was gently excised from the lower as well as upper lip, and fat was subsequently stripped off. Eventually, the underlying muscle tissue was sliced off until only a thin sheet remained to provide space for intramucosal injections. The tissue sample was divided into 2 × 2 cm pieces for injection of pemphigus autoantibodies and the p38MAPK inhibitor used in the study. A larger piece of the specimen was required as the tissue is very delicate, and hence areas grasped with tweezers were carefully removed to avoid any damage incurred from handling (Figure 6(g)i). A 30G syringe was allowed to pass through the mucosa sample along its long axis (Figure 6(g)ii). Then, either pathogenic monoclonal Dsg3 pemphigus autoantibody AK23 or mdPV-IgG was injected into the sample. Controls were injected with IgG from a healthy volunteer. In the case of p38MAPK inhibition, samples were treated with different concentrations of SB202190. Injection areas were marked (Figure 6(g)iii) and samples were allowed to float on DMEM with the mucosa facing upwards without any additional support, then incubated at 37°C and 5% CO_2_ for indicated time periods (Figure 6(g)iv).

This new ex vivo human mucosal model was validated through the evaluation of Dsg and desmocollin (Dsc) isoforms distribution in mucosa control (Figure 6(h)) which represent the homogenous distribution of Dsg3 antibodies throughout the oral mucosa. The viability and sign of apoptosis was also investigated in the ex vivo mucosal model (Figure 6(i)) and no signs of apoptosis was detected and the viability of the mucosal tissue was also intact in presence or absence of p38MAPK inhibitor SB202190 (Figure 6(j)). However, following AK23 and mdPV-IgG injection blister formation was evident in all the samples (Figure 6(k)(i–iii)) and even the samples pretreated with p38MAPK inhibitor SB202190 showed the same extent of blister formation as those treated with AK23 or mdPV-IgG alone. These results show that PV-IgG and AK23 induced blisters and desmosome ultrastructural changes are not dependent on p38MAPK dependent signalling pathway.

##### *ex vivo* model for infected root canal

Furthermore, to investigate the bacterial extrusion from infected root canals during masticatory function have also been investigated using an ex vivo model.^268^ In this model 12 extracted human single-rooted anterior teeth with complete root formation were selected for having comparable root canal space and apical foramina by using periapical radiography. The teeth were completely free from restorations with no previous endodontic treatment. The teeth were autoclaved before use and then stored in sodium azide solution. All teeth were subject to periapical radiographs in the buccolingual and mesiodistal planes. The total two-dimensional area of the root canal space in both projections, as well as the width of the apical foramina, was measured. Five teeth were allocated to the occlusal loading group and five to the static group (no loading). Endodontic access preparations were prepared, and the working length of the canals was established at 1 mm short of the apex. Root canal preparations were performed by using the Vortex rotary system to size 35 with a 0.06 taper, followed by irrigation with 2 ml of 5.25% NaOCl. The teeth were then irrigated and stored in 5% sodium thiosulfate for 1 min to inactivate the hypochlorite, and all the teeth were autoclaved. Twenty microlitres of one McFarland concentrations of S. intermedius bacterial suspension in thioglycolate broth was transferred to each of the root canals. The roots were incubated anaerobically for 21 days in 100% humidity to allow colonisation of the bacteria on the canal wall and into the dentinal tubules. A similar concentration of bacterial suspension of the bacteria was added every week. After 21 days of inoculation, all samples were loaded to the test apparatus.

The apparatus (Figure 7(a)) consisted of a modified plastic vial with two chambers separated by a thin built-in diaphragm with a small opening created to allow 3 mm of the tooth root to suspend into the lower chamber containing dental transport medium. The upper chamber of the vial was filled with polyvinyl siloxane (PVS) (to mimic the periodontal ligament) so that the crown is exposed, and the root surface from the cementoenamel junction to a level 3 mm short of the apex is enclosed by the PVS. The five experimental teeth were then mounted into their respective apparatuses. Once the lower chamber was filled with dental transport medium, a plastic screw cap at the base of the plastic tube of each apparatus was tightened to secure closure (Figure 7(b)). The apparatuses were mounted to the lower sample holder of a chewing simulator. After a simulated chewing equivalent to 1 year of function (240,000 cycles over 3 days) at a dynamic force of 117 N for the experimental dynamic loading group, 0.5 ml aliquots of the respective bacterial transport media were collected from the lower chamber of all specimens (static and dynamic) by aspirating through the bottom of the plastic screw cap Quantitative bacterial presence in the bottom chamber was assessed by counting the CFUs. The result demonstrated a significantly higher CFUs in the dynamic group, whereas the static group showed much lower growth. Moreover, the static group showed no periapical bacterial extrusion and there was no statistically significant interaction between the type of bacteria and chewing simulation.

##### *ex vivo* modelling for oral mucosal reconstruction

Oral and Maxillofacial surgeons are often confronted with the need for a mucosal lining for oral reconstruction after trauma, surgical resection, or pre-prosthetic surgery. Mucosal reconstruction of the oral cavity has involved the use of either split-thickness skin grafts (STSG) or palatal oral mucosal grafts, both of which require a second surgical procedure. The STSG has a distinct disadvantage in its texture, and the palatal oral mucosa is limited in supp1y^269^ which encourages the production of oral mucosa equivalents. The production of an oral mucosa equivalent consists of two steps: Keratinocyte cultivation (primary cell culture) and the production of the oral mucosa equivalent on a scaffold. In the past, the technique of Rheinwald and Green has been used to fabricate cultured oral epithelial sheets. Their protocol uses a feeder layer composed of irradiated 3T3 mouse fibroblasts to grow keratinocytes in vitro.^270,271^ However, these models are undesirable in elective surgery because of the undetermined risk of introducing a high mouse DNA content onto proliferating human cells.^272^ Previous studies have shown that the use of a composite containing a dermal matrix can assist in epithelial graft adherence and maturation and minimise wound contracture.^273,274^ Unfortunately, oral mucosal equivalents composed of a dermal matrix of type I collagen and populated with fibroblasts have also been shown to be fragile, difficult to handle and needs Vaseline gauze to carry the composite to the surgical site or wound.^275,276^

In this scenario, Izumi et al.^277^ demonstrated that AlloDerm (LifeCell Co, Woodlands, TX) may be an excellent choice of dermal matrix for the development of oral mucosal equivalent. AlloDerm is an acellular, nonimmunogenic cadaveric human dermis.^278^ It has a polarity by which one side of the material has a basal lamina to grow epithelial cells on, and the other side, an underlying porous dermal matrix, allows in growth of fibroblasts and angiogenic cells.^279^ In addition, the AlloDerm has handling characteristics that would allow for the application and stabilisation of the oral mucosal composite within the oral cavity. In this study Billingham and Reynolds have developed a composite human oral mucosal equivalent without the use of an irradiated mouse feeder layer and in a defined culture medium, which may be suitable for intraoral grafting procedures. To produce this ex vivo composite firstly AlloDerm was cut into circular pieces with a 11.3 mm diameter to conform to the area of a 48-well micro plate. The circular samples were rehydrated in phosphate buffered saline without Ca^+^ and Mg^++^ and placed into the 48-well culture plate with 100 µl FBS for 1 h to enhance attachment of seeded keratinocytes. The keratinised oral tissue samples from masticatory mucosa were collected from patients having dental extractions, pre-prosthetic surgeries and gingivectomies. The oral mucosal keratinocytes were cultivated using enzymatic method.^280^

Oral keratinocytes from the second or third passage of actively dividing cells were used to seed the AlloDerm and harvested by first washing with solution ‘A’ (30 mmol/l hydroxyethylpiperazine-N’-2-ethanosulfonic acid [HEPES], 10 mmol/l glucose, 3 mmol/L KCl, 130 mmol/l NaCI, 1.0 mmol/l Na_2_HPO_4_, pH 7.4) followed by addition of a solution of trypsin-EDTA. Trypsin activity was inhibited by soybean tissue inhibitor. Disaggregated cells were collected, counted, centrifuged and resuspended. Cells of different concentrations were placed on l cm^2^ pieces of AlloDerm and incubated with MCDB 153 medium, containing a high concentration of calcium. After 6 h the medium was changed to remove the added FBS. The oral keratinocyte-AlloDerm composites were cultured submerged for 4 days in the 48-well culture plate. After incubating the composites, they were transferred to an organotypic tissue culture flask and allowed the composites to grow at an air-liquid interface. Which encourages stratification of epithelial layer. The rehydrated AlloDerm showed no evidence of any cellular components (Figure 7(c)) and consisted of interlacing dense collagen bundles of varying sizes, indicating that the structural integrity of the ECM was intact. At day 11, after 1 week of being raised to the air-liquid interface, a continuous epithelial layer was entirely generated on the epidermal surface (Figure 7(d)).

Nevertheless, several technical obstacles in the enzymatic method can be encountered, such as long culture periods (3–4 weeks) and low intake rates in the enzymatic treatment.^281,282^ In 1910, Carrel and Burrows^283^ described a method for the extraction of epithelial cells called direct explant, which has been used since that time. The direct explant technique has also been used for 30 years in the culturing of human oral tissues.^284,285^ In the direct explant technique, the cells from the initial tissue extraction affixed to a culture dish migrate out of the tissue starting from the edges of the fragment, adhering to and multiplying on the culture dish. It has been suggested that direct explant technique is more successful than the enzymatic technique in culturing human oral keratinocytes.^286,287^ Thus, Bayar et al. developed an optimised version of the original direct explant to obtain keratinocyte culture and produce ‘ex-vivo produced oral mucosa equivalents’ (EVPOME). In this study, the oral mucosal keratinocytes were cultivated by direct explant technique where each tissue specimen were cut into 8–10 pieces, approximately 1 mm × 1 mm in size, and placed in the different culture flasks followed by incubation with culture media (DMEM supplemented with 10% heat-inactivated foetal calf serum (FCS), 100 U/ml penicillin, 100 μg/ml streptomycin and 0.5% amphotericin B (Figure 7(e)). Following keratinocyte culture, the cells were then decorated on AlloDerm and composite of keratinocytes on Alloderm was developed.^277^

This method of EVPOME production depicted 100% success in keratinocyte cultivation along with that the average number of days required for epithelial cells to grow and migrate out from the tissue origin was around 9–10 days and it took a total of 20 days for the cells to become fully confluent. Moreover, histological evaluation of ‘EVPOME’s showed multilayered epithelium comprising basal, suprabasal, and parakeratinised layers. The epithelial architecture of ‘EVPOME’s resembled that of normal oral mucosa. Highly stratified ‘EVPOME’s showed evidence of parakeratosis. Keratinocytes of the basal layer were cuboid, and they were aligned along the ‘AlloDerm’ surface. Under the basal layer of ‘EVPOME’s, ‘AlloDerm’ showed no evidence of any cellular components and consisted of interlacing dense collagen bundles of varying sizes, indicating that the structural integrity of the ECM was intact. The thickness of the epithelial sheet was measured to be 3–7 cell-layers in ‘EVPOME’ and 7–20 cell-layer in the original tissue sample (Figure 7(f)).

### Limitations of *ex vivo* modelling

Although ex vivo models provide a cheap and high-throughput alternative to in vivo models, they share some common limitations. Based on the above-mentioned scientific literature, it can be concluded that ex vivo models are optimal for both the analysis of drug permeability and for reducing the number of in vivo experiments; however, due to atherogenicity related to tissue origin and preparation, these models cannot completely replace in vivo tests, even if more recent publications are making progress in standardising and automating many experimental procedures.^288,289^ In addition, similar to in vitro models, one of the major disadvantages of the ex vivo model is the lack of natural immune systems. Migration of cells from blood into the experimental tissue during immune responses cannot be assessed. Culturing conditions in ex vivo models can also deviate from the natural environment found in animal models, although synthetic media has been developed to mimic the native environment. Finally, the lifespan of ex vivo models is often limited compared with the timespan of chronic infections.
